# Variational quantum metrology with the Loschmidt echo

**DOI:** 10.1093/nsr/nwaf091

**Published:** 2025-03-10

**Authors:** Ran Liu, Ze Wu, Xiaodong Yang, Yuchen Li, Hui Zhou, Zhaokai Li, Yuquan Chen, Haidong Yuan, Xinhua Peng

**Affiliations:** CAS Key Laboratory of Microscale Magnetic Resonance and School of Physical Sciences, University of Science and Technology of China, Hefei 230026, China; Anhui Province Key Laboratory of Scientific Instrument Development and Application, University of Science and Technology of China, Hefei 230026, China; Institute of Quantum Precision Measurement, State Key Laboratory of Radio Frequency Heterogeneous Integration, College of Physics and Optoelectronic Engineering, Shenzhen University, Shenzhen 518060, China; CAS Key Laboratory of Microscale Magnetic Resonance and School of Physical Sciences, University of Science and Technology of China, Hefei 230026, China; Anhui Province Key Laboratory of Scientific Instrument Development and Application, University of Science and Technology of China, Hefei 230026, China; Institute of Quantum Precision Measurement, State Key Laboratory of Radio Frequency Heterogeneous Integration, College of Physics and Optoelectronic Engineering, Shenzhen University, Shenzhen 518060, China; Quantum Science Center of Guangdong-Hong Kong-Macao Greater Bay Area (Guangdong), Shenzhen 518045, China; CAS Key Laboratory of Microscale Magnetic Resonance and School of Physical Sciences, University of Science and Technology of China, Hefei 230026, China; Anhui Province Key Laboratory of Scientific Instrument Development and Application, University of Science and Technology of China, Hefei 230026, China; School of Physics, Hefei University of Technology, Hefei 230009, China; CAS Key Laboratory of Microscale Magnetic Resonance and School of Physical Sciences, University of Science and Technology of China, Hefei 230026, China; Anhui Province Key Laboratory of Scientific Instrument Development and Application, University of Science and Technology of China, Hefei 230026, China; Hefei National Laboratory, University of Science and Technology of China, Hefei 230088, China; CAS Key Laboratory of Microscale Magnetic Resonance and School of Physical Sciences, University of Science and Technology of China, Hefei 230026, China; Anhui Province Key Laboratory of Scientific Instrument Development and Application, University of Science and Technology of China, Hefei 230026, China; Department of Mechanical and Automation Engineering, The Chinese University of Hong Kong, Hong Kong SAR, China; CAS Key Laboratory of Microscale Magnetic Resonance and School of Physical Sciences, University of Science and Technology of China, Hefei 230026, China; Anhui Province Key Laboratory of Scientific Instrument Development and Application, University of Science and Technology of China, Hefei 230026, China; Hefei National Laboratory, University of Science and Technology of China, Hefei 230088, China

**Keywords:** quantum metrology, Loschmidt echo, variational quantum optimization, quantum Fisher information

## Abstract

By leveraging quantum effects, such as superposition and entanglement, quantum metrology promises higher precision than classical strategies. It is, however, a challenging task to achieve the higher precision on practical systems. This is mainly due to difficulties in engineering nonclassical states and performing nontrivial measurements on the system, especially when the number of particles is large. Here we propose a variational scheme with the Loschmidt echo for quantum metrology. By utilizing hardware-efficient ansatzes in the design of variational quantum circuits, the quantum Fisher information (QFI) of the probe state can be extracted from the experimentally measured Loschmidt echo in a scalable manner. This QFI is then used to guide the online optimization of the preparation of the probe state. We experimentally implement the scheme on an ensemble of 10-spin quantum processors and achieve a 12.4-dB enhancement of the measurement precision over the uncorrelated states, which is close to the theoretical limit. The scheme can also be employed on various other noisy intermediate-scale quantum devices, which provides a promising protocol to demonstrate quantum advantages.

## Teaser

This study introduces a variational quantum metrology scheme based on Loschmidt echo and experimentally demonstrates its quantum-enhanced characteristic, providing a promising framework for quantum metrology across diverse practical quantum systems.

## INTRODUCTION

To sense more accurately has always been one of the main drives for scientific advances and technological innovations. Quantum metrology [1–3], which utilizes quantum correlations to achieve higher sensitivities, has gained much attention recently. In ideal scenarios, quantum metrology can achieve a precision at the Heisenberg limit, which scales as $1/N$ with *N* the number of particles [4–7]. As a contrast, the precision of the classical strategies is bounded by the standard quantum limit (SQL), which scales as $1/\sqrt{N}$. To achieve higher precisions in quantum metrology, nontrivial entangled probe states, however, need to be prepared. This poses a practically challenging task when the number of particles increases. In practice, there are two main difficulties in achieving the highest precision. First, it is difficult to identify the optimal probe state when the number of particles increases. Because of the ‘curse of dimensionality’, the classical optimization that is required to identify the optimal probe state soon becomes intractable [8–11]. Second, it is a challenging task to prepare the identified optimal probe state on practical systems due to device-specific constraints, such as decoherences, imperfect controls and readout errors [12–14].

Variational quantum metrology (VQM) provides a promising route to circumvent these problems. In VQM the identification of the optimal probe state is carried out with a hybrid quantum-classical scheme. A variational quantum circuit is used to prepare the probe state and the circuit is optimized externally by a classical computer [15–18]. This hybrid scheme inherits the advantages of the variational quantum algorithm that not only reduces the complexity of the classical simulation, but can also easily incorporate the device-specific constraints into the design of the variational quantum circuit (VQC). The optimization of the circuit, however, can still be very challenging for quantum metrology. This is because the quantum Fisher information (QFI), which is often taken as the figure of merit in quantum metrology, is difficult to evaluate. The general brute-force approaches to extract QFI, such as quantum state tomography, demand an exponentially growing number of measurements [19]. Although some effective surrogates of QFI have been proposed previously, such as those based on additional physical qubits or experimental measurements [18,20–24], they may still require a considerable number of experimental measurements or extra physical qubits. This can go beyond current experimental capabilities.

In this article, we propose a variational optimization scheme for quantum metrology that uses the Loschmidt echo (LE) to efficiently extract the QFI. The signal of the LE can then be directly used to optimize the VQC that prepares the optimal probe state in quantum metrology. We demonstrate the power of the scheme by identifying and preparing a 10-spin optimal probe state in nuclear magnetic resonance (NMR) for the estimation of an unknown phase, where the system is in mixed states at room temperature. We experimentally implement the scheme and demonstrate that the achieved precision is close to the fundamental bound in quantum metrology—the quantum Cramér–Rao bound (QCRB). This opens a promising avenue for the implementation of quantum-enhanced parameter estimation on practical quantum devices due to its efficiency, robustness against experimental imperfections and easy implementation.

## RESULTS

### Scheme

We consider the iconic task of estimating parameter $\alpha$ in operator $U_\alpha ={\rm e}^{-i\alpha G}$ with *G* as the generator. The ultimate precision can be quantified by the QCRB, [1–3,25,26] as


**Figure 1.**
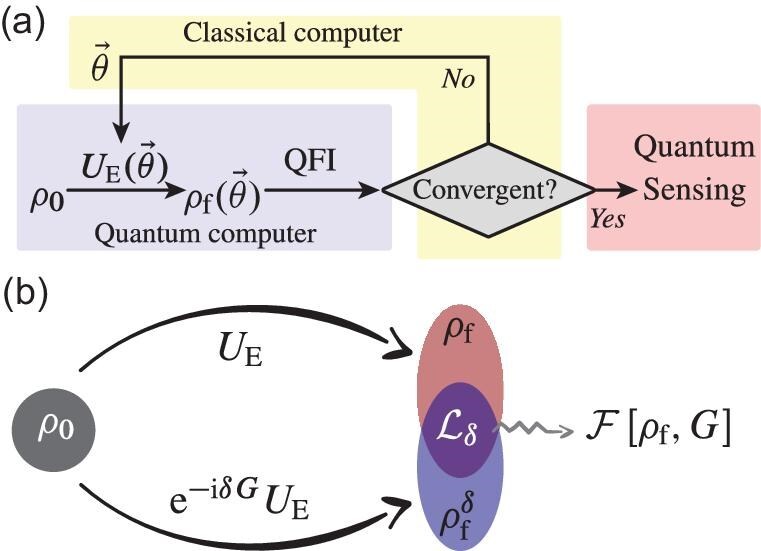
(a) Workflow for quantum probe engineering via quantum variational optimization. By taking the QFI as the figure of merit for the optimization of the VQC, the probe state is steered to the optimal state for high-precision phase estimation under practical dynamics. (b) Schematic diagram of measuring the LE. When the unperturbed evolution is specified as the engineering operation, i.e. $U\rightarrow {U}_{E}(\vec{\theta })$, and the perturbation in the perturbed evolution is specified as a small quench under encoding dynamics, i.e. ${U}_\delta \rightarrow {\rm e}^{-i\delta G}{U}_{E}(\vec{\theta })$, the QFI of the engineered probe ${\rho}_{f}$ can then be extracted from the LE.

(1) \begin{eqnarray*}
\Delta \alpha \ge \frac{1}{\sqrt{\nu \mathcal {F}}},
\end{eqnarray*}

where $\Delta \alpha$ is the standard deviation of an unbiased estimator $\hat{\alpha }$, $\nu$ is the number of repetitive measurements and $\mathcal {F}$ is the QFI. Our target here is to engineer a probe state with the maximal QFI, which leads to the smallest standard deviation. Here, the probe state is prepared by a VQC, which generates a unitary operation, ${U}_{E}(\vec{\theta })$, acting on a natural initial state of the physical system with $\vec{\theta }$ being the tunable parameters of the circuit. By taking the QFI as the figure of merit, we then optimize $\vec{\theta }$ to steer the probe state towards the optimal or nearly optimal state. This state is subsequently used for high-precision phase estimation. The schematic for the workflow of quantum probe engineering via VQM is illustrated in Fig. 1a.

An essential part of the variational optimization is to efficiently evaluate the figure of merit that determines how the parameters should be tuned. However, the standard methods of evaluating the QFI, such as state tomography, are extremely demanding in experiments. Here we develop an experimental protocol that uses the Loschmidt echo to evaluate the QFI.

For the pure state, the Loschmidt echo is given by $\mathcal {L}_\delta =|\langle \Psi _{0}|U^\dagger U_\delta | \Psi _{0}\rangle |^{2}$, which is the overlap between the states obtained from the forward unperturbed evolution $(U)$ and the forward perturbed evolution $(U_\delta )$. The Loschmidt echo corresponds to a susceptibility to the perturbation [27,28]. As shown in Fig. 1b, the Loschmidt echo can be used to extract the QFI when we substitute *U* and $U_\delta$ with ${U}_{E}(\vec{\theta })$ and ${\rm e}^{-i\delta G}{U}_{E}(\vec{\theta })$, respectively. In this case, the Fisher information can be evaluated from the Loschmidt echo as [29]


(2) \begin{eqnarray*}
\mathcal {F}[{U}_{E}(\vec{\theta }) |\Psi _0\rangle ]=\lim _{\delta \rightarrow 0}4\frac{1-\mathcal {L}_{\delta }}{\delta ^2}.
\end{eqnarray*}

We generalize this connection to the initially mixed quantum system, in which the considered process for state preparation is still unitary. The Loschmidt echo then becomes


(3) \begin{eqnarray*}
\mathcal {L}_\delta & \equiv& {\mathrm{Tr}}[{\rho}_{\mathrm{f}}{\rho}_{\mathrm{f}}^\delta ] \\
& \approx& \Gamma ({\rho}_{\rm f})\!-\!\frac{\delta ^2}{4} \left[\!2\sum \limits_{i,j=1}^d(\lambda _i-{\lambda}_ {j})^2|\langle {\psi}_{i|G|}{\psi}_{j}\rangle |^2\!\right]\!.\!\! \\
\end{eqnarray*}

Here ${\rho}_{\mathrm{f}}={U}_{E}(\vec{\theta }){\rho}_{0}{U}_{E}^\dagger (\vec{\theta }) =\sum _{i=1}^d\lambda _i|\psi _i\rangle \langle \psi _i|$, ${\rho}_{\rm f}^\delta ={\rm e}^{-i\delta G}{U}_{E}(\vec{\theta }){\rho}_0{U}_{E}^{\dagger} (\vec{\theta }){\rm e}^{i\delta G}$, *d* is the dimension of the Hilbert space and $\Gamma ({\rho}_{\rm f})=\sum _{i=1}^d\lambda _i^2 $ is the purity of the state, which in our case can be treated as a constant since it does not change under unitary evolution. The LE is connected to the QFI of mixed states as (see the Methods section below for detailed deviations)


(4) \begin{eqnarray*}
\mathcal {F}[\rho _{\rm f},H]\ge \lim _{\delta \rightarrow 0}4\frac{\Gamma (\rho _{\rm f})-\mathcal {L}_\delta }{\delta ^2}.
\end{eqnarray*}

Though only a lower bound on QFI can be extracted from this inequality, this bound is directly related to sub-QFI, which shares the same global extrema with QFI [22] and can thus be employed in the variational optimization of the probe state. For highly mixed states where the eigenvalues are almost degenerate, i.e. $\lambda _i\approx {1}/{d}$ for $1\le i\le d$, the bound can also be saturated with


(5) \begin{eqnarray*}
\mathcal {F}[\rho _{\rm f},H]\approx \lim _{\delta \rightarrow 0}2d\frac{\Gamma (\rho _{\rm f})-\mathcal {L}_\delta }{\delta ^2}.
\end{eqnarray*}

This is exactly the case in NMR as the initial state of the NMR system is a thermal state with the Boltzmann distribution, which at room temperature is close to the completely mixed state [30]. Since $\rho _{\rm f}={U}_{E}(\vec{\theta })\rho _0{U}_{E}^\dagger (\vec{\theta })$ has the same eigenvalue as $\rho _0$, $\rho _{\rm f}$ is thus also almost degenerate.

For a better understanding of the experimental extraction of the LE, we rewrite Equation (3) as


(6) \begin{eqnarray*}
\mathcal {L}_\delta \equiv {\rm Tr} [V_\delta (\vec{\theta })\rho _0V_\delta ^\dagger (\vec{\theta })\rho _0]
\end{eqnarray*}

with $V_\delta (\vec{\theta })\equiv {U}_{E}^\dagger (\vec{\theta }){\rm e}^{-i\delta G}{U}_{E}(\vec{\theta })$. The LE can thus be obtained by first using the variational quantum circuit to generate ${U}_{E}(\vec{\theta })$, then applying a perturbation evolution ${\rm e}^{-i\delta H}$, followed by a backward evolution $U^\dagger _{\rm E}(\vec{\theta })$ and a projection onto the initial state.

We note that the initial states of practical quantum systems are typically classical product states, making the LE efficiently extractable from linearly increasing local measurements with the system size and experimentally favorable. Moreover, the VQCs can be designed with hardware-efficient ansatzes [31], which not only enhance their feasibility across diverse quantum systems by accommodating the constraints of current quantum hardware, but also ensure that the backward evolution $U^\dagger _{\rm E}(\vec{\theta })$ can be implemented in a scalable manner (see the online supplementary material).

### Experimental variational optimization of the 10-spin mixed quantum probe state

**Figure 2.**
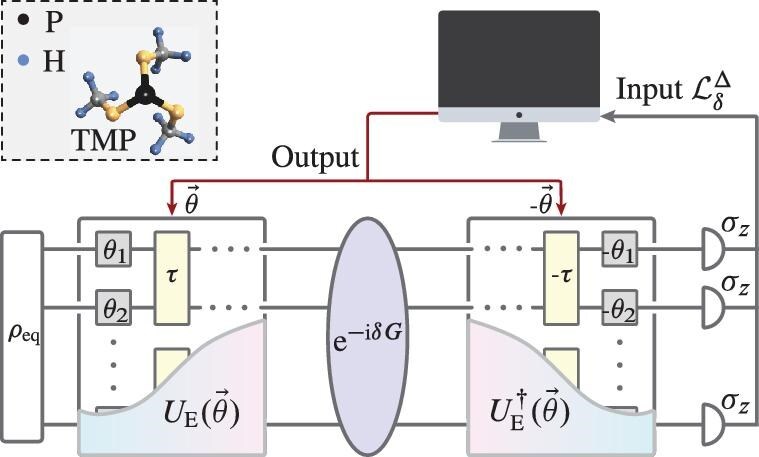
The experimental procedures for variationally optimizing the metrologically useful mixed state via the LE. The 10-spin quantum probe is realized by a $^{31}{\rm P}$ nuclear spin and nine equivalent $^1 {\rm H}$ spins in the TMP molecule and initialized as an equilibrium state $\rho _{\rm eq}$. Here $\rho _{\rm eq}$ evolves under the symmetrical variational quantum circuit $V_\delta (\vec{\theta })\equiv {U}_{E}^\dagger (\vec{\theta }) {\rm e}^{-i\delta G}{U}_{E}(\vec{\theta })$ and the polarization of each spin along the *z* axis is then measured to obtain the LE $\mathcal {L}_\delta$. The QFI of the quantum probe, i.e. $\mathcal {F}[\rho _{\rm f}(\vec{\theta }),G]$, can be extracted from the LE and feedback to the classical computer, which is employed to iteratively update parameters $\vec{\theta }$ to maximize the QFI.

We experimentally demonstrate the scheme on a Bruker Avance III 400-MHz NMR spectrometer at room temperature. The sample is trimethylphosphite (TMP) dissolved in $d_6$ acetone. The TMP molecule, which consists of a central $^{31} {\rm P}$ nuclear spin and nine equivalent $^{1} {\rm H}$ nuclear spins, as shown in Fig. 2, is employed as the 10-spin quantum probe. In the liquid state, the interaction between $^{1} {\rm H}$ spins is negligible due to the magnetic equivalence. The natural Hamiltonian of the system in the doubly rotating frame is $H_{\rm {NMR}}={\pi }J_{\rm {PH}}\sigma _z^1 / 2\otimes \sum _{j=2}^{10}\sigma _z^j$ with $J_{\rm PH}=10.5$ Hz. Here we use Arabic numerals 1–10 to respectively denote the $^{31} {\rm P}$ nuclear spin and nine $^1 {\rm H}$ nuclear spins.

Figure 2 shows the experimental procedures for engineering the mixed probe state via the hybrid quantum-classical scheme with quantum variational optimization. Here the extraction of the LE is performed on the quantum system, while the updating of the parameters is determined on the classical computer. The quantum part contains three major stages as described below.

1. The system is initially in the uncorrelated equilibrium state at room temperature, ${\rho}_{\rm eq}=(\mathbb{1} + \epsilon {\rho}_{\rm eq}^\Delta )/2^{10}$, where $\rho _{\rm eq}^\Delta =\sum _{j=1}^{10}\gamma _{j}\sigma _z^{j}/2 $, $\mathbb{1}$ is the $2^{10}\times 2^{10}$ unit operator, $\epsilon$ is the thermal polarization ($\sim\! 10^{-5}$) and $\gamma _{j}$ is the relative gyromagnetic ratio of the corresponding nuclear spin with $\gamma _1=0.8,\gamma _{2,3,\dots ,10}=2.0$.
2. Evolve the system under $V_\delta (\vec{\theta })\equiv {U}_{E}^\dagger (\vec{\theta }) {\rm e}^{-i\delta G}{U}_{E}(\vec{\theta })$ according to Equation (6). In our experiment, ${U}_{E}(\vec{\theta })$ is realized by a three-layer VQC consisting of single-spin rotations, i.e. ${\rm e}^{-i\theta _k\sigma _{x,y}/2}$ with $\vec{\theta }\equiv (\theta _1,\theta _2,\dots ,\theta _k,\dots )$, and the free evolution under Hamiltonian $H_{\rm NMR}$ for a duration $\tau$. The interactions in $H_{\rm NMR}$ facilitate the generation of nonclassical correlations in the probe state, thereby enabling the potential to achieve precision beyond the SQL. Details of the VQC can be found in the online supplementary material. After the preparation of the optimal probe state, the dynamics that encodes parameter ${\rm e}^{-i\delta G}$ is then applied. Without loss of generality, we consider the encoding dynamics as a field along the *z* axis, and the corresponding Hamiltonian is $G=\sum _{k=1}^{10}\sigma _z^j/2$. Theoretically, the correspondence between the Loschmidt echo and QFI is best when $\delta \rightarrow 0$, as indicated in Equation (5). However, the experiment signal of the Loschmidt echo is least sensitive to the change of the parameter when $\delta =0$ since $\mathcal {L}_\delta =\Gamma (\rho _f)$ reaches the maximal where the derivative is zero. So there exists a trade-off. With the aid of numerical simulation, we find that $\delta =0.2$ is optimal for our experiment (see the online supplementary material). Finally, the reverse evolution $U^\dagger _{\rm E}(\vec{\theta })$ is performed. This can be implemented by applying the reverse evolution of each operation in the PQC in reverse order. Specifically, the reverse of single-spin rotations can be implemented by changing the phase of each pulse, and the reverse of the free evolution under $H_{\rm NMR}$ can be implemented by applying $\pi$ pulses along the $x $ direction to the $^{31}$P spin at both the beginning and end of the evolution.
3. Project the evolved state onto the initial state $\rho _0$. Substituting the specific form of $\rho _0$ into Equation (6), we have
  (7) \begin{eqnarray*}
\mathcal {L}_\delta &=&\frac{1}{2^N} + \frac{\epsilon }{2^N}\sum _{j=1}^{10}\gamma _j {\rm Tr}\\
&&\left[V_\delta (\vec{\theta })\rho _0V_\delta ^\dagger (\vec{\theta })\sigma _z^j\right].
\end{eqnarray*}
  This means that the LE can be extracted from the local measurement of the evolved state $V_\delta (\vec{\theta })\rho _0V_\delta ^\dagger (\vec{\theta })$, i.e. the polarization of each spin along *z* axis. Hence, the measurement overhead increases linearly with the system size. The identity $\mathbb{1}$ in $\rho _0$ does not change under the unitary evolution $V_\delta (\vec{\theta })$ and also does not contribute to the experimental signal since the observables in NMR are traceless. The Loschmidt echo in Equation (7) then becomes
  (8) \begin{eqnarray*}
\mathcal {L}_\delta =\frac{1}{2^N}+\frac{\epsilon ^2}{2^{2N}}\mathcal {L}_\delta ^\Delta
\end{eqnarray*}
  with $\mathcal {L}_\delta ^\Delta \equiv \sum _{j=1}^{10}\gamma _j{\rm Tr} (V_\delta (\vec{\theta })\rho _{\text eq}^\Delta V^\dagger _\delta (\vec{\theta })\sigma _z^j)$, and ${\rm Tr}(V_\delta (\vec{\theta })\rho_{\text eq}^\Delta V^\dagger _\delta (\vec{\theta })\sigma _z^j)$ directly obtained from the experimental measurements on different nuclear spins.

To reduce errors in measuring the LE, we employ several techniques in our experiment. We use single-spin rotations with the BB1 composited sequence [33] to address pulse shape imperfections. To enhance the signal-to-noise ratio, protons are decoupled during measurement of the $^{31}\text{P}$ nucleus signals. The total evolution duration is 19 ms, whereas the decoherence time is 44 ms. The signal decay due to decoherence is therefore non-negligible. We compensate for this decay by calibrating the signal $\mathcal {L}_\delta ^{\Delta }$ using $\mathcal {L}_0^\Delta \equiv \sum _{j=1}^{10}\gamma _j{\rm Tr}(V_0(\vec{\theta })\rho _{\text eq}^\Delta V^\dagger _0(\vec{\theta })\sigma _z^j)$, which has a known theoretical value and a similar level of decay as $\mathcal {L}_\delta ^{\Delta }$ (see the online supplementary material).

With the extracted LE from our quantum processor, we proceed to train the parameters in the PQC using a classical optimizer. Specifically, we adopt the Nelder–Mead (NM) algorithm [34] due to its enhanced robustness against noise and ability to explore neighboring valleys to identify better local optima. These characteristics make the NM algorithm particularly well suited for our experimental implementation. We have also made modifications to the algorithm to further improve its efficiency (see the online supplementary material).

**Figure 3.**
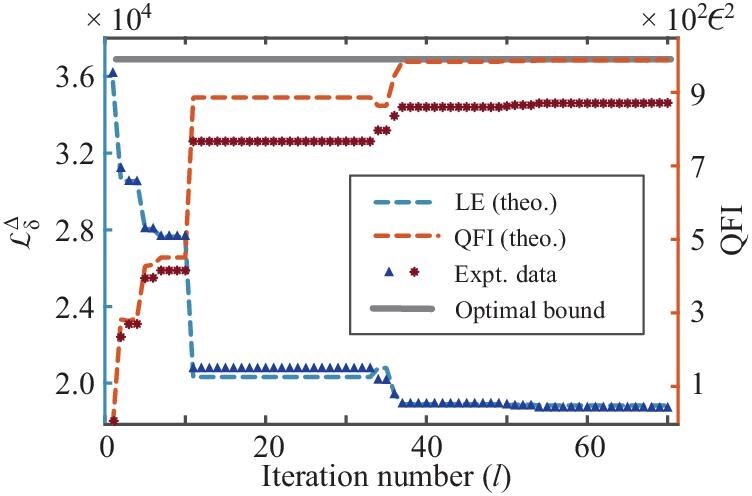
Experimental results of variational quantum optimization. The blue triangles are the measured $\mathcal {L}_\delta ^{\Delta }$ in the experiment. Error bars are absent due to the fluctuations being smaller than the size of the points (see the online supplementary material). The blue dashed line is the theoretical LE obtained from numerical calculation. The estimated QFI according to Equation (5) is depicted with red stars, while the red dashed line is the theoretical QFI. The theoretical maximum of the QFI given by Fiderer *et al.* [32] is $\mathcal {F}_{\text max}=989\epsilon ^2$ and plotted with a black solid line. The finally engineered probe is close to the optimal one even in the presence of experimental imperfections. To compensate for the signal decay caused by relaxation, the experimental results of the LE and QFI have been calibrated.

The experimental results are illustrated in Fig. 3, where the blue triangles represent the measured $\mathcal {L}_\delta ^{\Delta }$ obtained in the experiment. It is observed that the signal initially drops rapidly and tends to stabilize with increasing iteration number *l*, capped at a maximum of 70. The blue dashed line shows the theoretical LE signal, serving as a benchmark for experimental accuracy. The relative error between the theoretical and experimental results is $1.35\%$, primarily attributed to relaxation effects. A detailed analysis of the experimental error is given in the online supplementary material. The QFI extracted from the experimental data using Equation (5) is represented with red stars, while the theoretical QFI is represented with a red dashed line. The discrepancy between them arises from the experimental errors and the neglected higher-order terms in the correspondence between the Loschmidt echo and QFI at finite $\delta$. Additionally, the optimal QFI predicted by Fiderer *et al.* [32] is plotted with a solid black line. While the theoretical QFI does not always increase as the experimental one, for instance at $l=34$ due to experimental error, it still converges to a significantly enhanced QFI close to the maximum. This result validates the feasibility of our scheme in the presence of experimental imperfections.

### Phase estimation

To demonstrate the enhanced precision of our engineered mixed probe in quantum parameter estimation, we apply it to a typical quantum metrology application—quantum phase estimation [35–37]. While our earlier results indicate that the engineered probe state via variational optimization exhibits a significantly improved QFI, approaching the ultimate QCRB given by the optimized QFI necessitates an optimal or near-optimal readout protocol. Here we adopt an easily implementable measurement protocol known as the time-reversal-based readout (TRBR) protocol, which exploits time-reversal dynamics to disentangle probe states for feasible readout and has been previously demonstrated on diverse platforms such as cold-atom cavity-QED systems [38], Bose–Einstein condensates [39] and trapped ions [40]. In our experiment, we employ the previously optimized state $\rho _{\rm f}$ as the probe and encode the parameter to be estimated $\alpha$, i.e. $\rho _{\rm f}^{\alpha }={\rm e}^{-i\alpha G}\rho _{\rm f}{\rm e}^{i\alpha G}$. To implement the TRBR protocol, we apply the inverse evolution $U_{\text{E}}^\dagger (\vec{\theta })$ before projecting onto the initial state $\rho _0$, which is equivalent to applying a near-optimal measurement $\mathcal {O}_{\text rev}=U_{\text{E}}(\vec{\theta })\rho _0U_{\text{E}}^\dagger (\vec{\theta })$ on $\rho _{\rm f}^\alpha$. Finally, we assess the performance of the optimized probe under the TRBR protocol according to the error propagation formula [4]


(9) \begin{eqnarray*}
(\Delta \alpha _{\rm f})^2=\frac{(\Delta \mathcal {O}_{\text rev})^2}{(d\langle \mathcal {O}_{\text rev}\rangle /d\alpha )^2},
\end{eqnarray*}

where $(\Delta \mathcal {O}_{\text rev})^2=\langle \mathcal {O}_{\text rev}^2\rangle -\langle \mathcal {O}_{\text rev}\rangle ^2$ represents the quantum fluctuation of $\mathcal {O}_{\text rev}$, and $\langle \mathcal {O}_{\text rev}\rangle ={\rm Tr}(\rho _{\rm f}^{\alpha }\mathcal {O}_{\text rev})$. Details of the experimental extraction of $(\Delta \alpha _{\rm f})^2$ are elaborated in the Methods section below.

**Figure 4.**
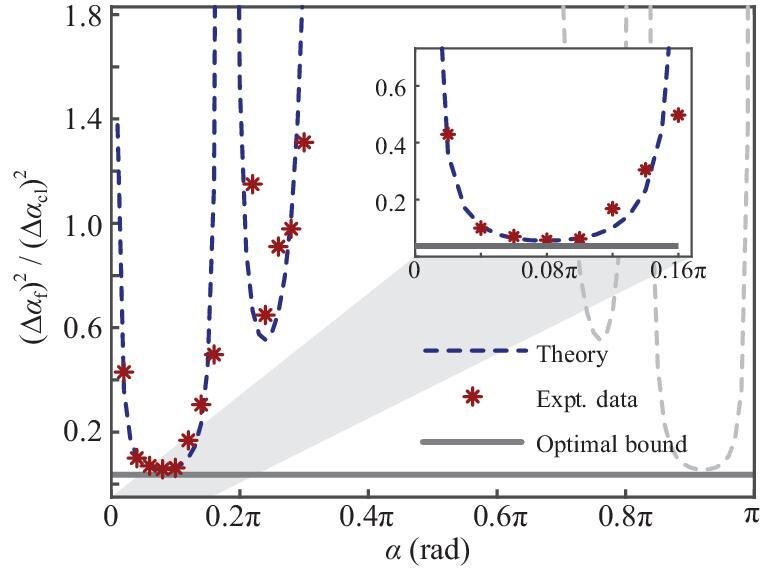
The precision ratio of the experimentally engineered state $\rho _{\rm f}$ to the classical uncorrelated state $\rho _{\rm cl}$. These experimental results have been calibrated to compensate for the signal decay caused by relaxation. The red stars are obtained from experimental measurements of $\rho _{\rm f}$, which outperforms the precision limit of $\rho _{\rm cl}$ by a factor of 12.4 dB (a factor of 10.7 dB without signal compensation), and the blue dashed line is the theoretical result. The gray solid line is the optimal precision given by the QFI and bounds the precision of the experimental measurements. It is more clear in the inset that though the time-reversal-based readout protocol is suboptimal, a precision close to the optimal QCRB can still be realized with the current engineered probe.

We benchmark the precision of the optimized mixed state $\rho _{\rm f}$ against its classical counterpart $\rho _{\rm cl}$, where $\rho _{\rm cl}$ is generated by local operations on individual spins from $\rho _0$ with a SQL-like precision scaling $\Delta \alpha _{\text cl}\sim 1/\sqrt{N}$ [41]. The experimental result of $(\Delta \alpha _{\rm f})^2/(\Delta \alpha _{\rm cl})^2$ is depicted in Fig. 4 with red stars, closely matching the theoretical prediction indicated by the blue dashed line. Under the experimental condition $\epsilon \sim 10^{-5}$, we have $\Delta \alpha _{\text cl}\sim 1.7\times 10^4$, and the optimum of $\Delta \alpha _{\rm f}$ occurs around $\widetilde{\alpha }=0.08\pi$, being $\Delta \alpha _{\rm f}\sim 4.0\times 10^{3}$. This results in a precision ratio $(\Delta \alpha _{\rm f})^2/(\Delta \alpha _{\text cl})^2=0.056$, corresponding to a 12.4-dB improvement. This improvement in precision, greater than $\sqrt{N}$, is attributed to the complex eigenspectrum of mixed states, as discussed by Modi *et al.* [41] and further detailed in the online supplementary material. In practical implementations, we can asymptotically approach this local precision by adaptively adjusting $\alpha$ near $\widetilde{\alpha }$ with an additional control field [42]. The QCRB is also plotted with a black solid line. The inset clarifies that while the TRBR protocol is suboptimal, the current engineered probe can still achieve quantum-enhanced precision close to the QCRB.

## CONCLUSIONS

To summarize, we propose a novel scheme for variational quantum metrology with the LE. We demonstrate its feasibility by engineering an optimal 10-spin mixed probe state on an NMR system, where the QFI is efficiently estimated using the LE to guide the variational optimization. By utilizing the proposed time-reversal-based readout protocol, the engineered probe achieves a quantum-enhanced precision that approaches the optimal quantum Cramér–Rao bound.

The proposed variational scheme features several advantages for experimental implementation. First, since the measured Loschmidt echo provides a faithful lower bound for QFI [22], this scheme can be extended to quantum systems with different purities. In addition, the scheme does not require detailed knowledge of the encoding dynamics during optimization, which is often unknown in practice. Furthermore, by utilizing VQCs designed with hardware-efficient ansatzes and a measurement overhead that scales linearly with system size, our scheme demonstrates practical efficiency and scalability for extracting QFI. This work paves the way for broader applications of variational quantum metrology to diverse quantum sensing tasks and quantum systems. Future work could explore the use of gradient-based classical optimizers to enhance efficiency. For example, the parameter-shift rule [43] enables direct gradient evaluation on quantum processors, potentially improving optimization in complex parameter spaces. We also anticipate that future research will explore our scheme on various NISQ computers [44–46], demonstrating quantum-enhanced precision.

## METHODS

### The connection between the Loschmidt echo and QFI

For the pure initial probe state $|\Psi _0\rangle$, the LE under engineering operation ${U}_{E}$ and encoding dynamics *G* can be expressed as


(10) \begin{eqnarray*}
\mathcal {L}_\delta =\left|\left\langle \Psi _0\left|{U}_{E}^\dagger {\rm e}^{-i\delta G}{U}_{E}\right|\Psi _0\right\rangle \right|^2.
\end{eqnarray*}

By expanding Equation (10) as a Taylor series around $\delta =0$, we have


\begin{eqnarray*}
\mathcal {L}_\delta &=&\langle \Psi _f|{\rm e}^{-i\delta G}|\Psi _f\rangle \langle \Psi _f|{\rm e}^{i\delta G}|\Psi _f\rangle \\
&=&\bigg (1-i\delta \langle G\rangle -\frac{\delta ^2}{2}\langle G^2\rangle +\frac{i\delta ^3}{6}\langle G^3\rangle \bigg )\\
&&\!\! \times \bigg (1\!+\!i\delta \langle G\rangle\! -\!\frac{\delta ^2}{2}\langle G^2\rangle\! -\!\frac{i\delta ^3}{6}\langle G^3\rangle \bigg )+O(\delta ^4)\\
&=&1-\delta ^2(\langle G^2\rangle -\langle G\rangle ^2)+O(\delta ^4),
\end{eqnarray*}

where $\langle \cdot \rangle \equiv \langle \Psi _f|\cdot |\Psi _f\rangle$ and $|\Psi _f\rangle \equiv {U}_{E}|\Psi _0\rangle$. As the QFI for a pure state is


(11) \begin{eqnarray*}
\mathcal {F}(|\Psi _f\rangle )=4(\langle G^2\rangle -\langle G\rangle ^2),
\end{eqnarray*}

we have [29]


(12) \begin{eqnarray*}
\mathcal {F}\left(|\Psi _f\rangle \right)=\lim _{\delta \rightarrow 0}4\frac{1-\mathcal {L}_{\delta }}{\delta ^2}.
\end{eqnarray*}

For the mixed engineered probe $\rho _{\rm f}={U}_{E}\rho _0{U}_{E}^\dagger$ with the eigendecomposition $\sum _{i=1}^d\lambda _i|\psi _i\rangle \langle \psi _i|$ and *d* as the dimension of the Hilbert space, the LE can be computed as


\begin{eqnarray*}
\mathcal {L}_\delta &=&{\rm Tr}(\rho _{\rm f} {\rm e}^{-i\delta G}\rho _{\rm f} {\rm e}^{i\delta G}) \\
&=&{\rm Tr}\left (\!\sum _{i=1}\lambda _i|\psi _i\rangle \langle \psi _i| {\rm e}^{-i\delta G}\sum _{j=1}\lambda _j|\psi _j\rangle \langle \psi _j| {\rm e}^{i\delta G}\!\right ) \\
&=&\sum _k\langle \psi _k|\sum _{i=1}\lambda _i|\psi _i\rangle \langle \psi _i| {\rm e}^{-i\delta G}\\
&&\times \sum _{j=1}\lambda _j|\psi _j\rangle \langle \psi _j| {\rm e}^{i\delta G}|\psi _k\rangle \\
&=&\sum _i\lambda _i^2-\delta ^2\Bigg(\sum _{i,j}\lambda _i\lambda _j|\langle \psi _i|G|\psi _j|^2\\
&& +\,\sum _i\lambda _i^2\langle \psi _i| G^2|\psi _i\rangle \Bigg)+O(\delta ^4).
\end{eqnarray*}

The zeroth-order term in the perturbation expansion, i.e. $\sum _i\lambda _i^2$, represents the purity of $\rho _0$, and it does not change under unitary transformation. However, for the second-order terms, note that


\begin{eqnarray*}
\sum _i\lambda _i^2\langle \psi _i|G^2|\psi _i\rangle &=&\frac{1}{2}\Bigg(\sum _i\lambda _i^2\langle \psi _i|G^2|\psi _i\rangle\\
&& +\, \sum _j\lambda _j^2\langle \psi _j|G^2|\psi _j\rangle \Bigg), \\
\langle \psi _i|G^2|\psi _i\rangle &=&\langle \psi _i|G\sum _j|\psi _j\rangle \langle \psi _j|G|\psi _i\rangle\\
&=&\sum _j|\langle \psi _i|G|\psi _j\rangle |^2;
\end{eqnarray*}

we thus have


\begin{eqnarray*}
\mathcal {L}_\delta &=&\sum _i\lambda _i^2+\sum _{i,j}\lambda _i\lambda _j\delta ^2|\langle \psi _i| G|\psi _j\rangle |^2 \\
&& -\frac{\delta ^2}{2}\sum _{i,j}\lambda _i^2\delta ^2|\langle \psi _i|G|\psi _j\rangle |^2 \\
&& -\frac{\delta ^2}{2}\sum _{i,j}\lambda _j^2\delta ^2|\langle \psi _i|G|\psi _j\rangle |^2+O(\delta ^4) \\
&=&\sum _i\lambda _i^2\!-\!\frac{\delta ^2}{4}\Bigg(2\sum _{i,j} (\lambda _i\!-\!\lambda _j)^2|\langle \psi _i|G|\psi _j\rangle |^2\Bigg).
\end{eqnarray*}

Comparing with the QFI for mixed states,


(13) \begin{eqnarray*}
\mathcal {F}(\rho _{\rm f})=2 \sum _{i, j} \frac{(\lambda _{i}-\lambda _{j})^{2}}{\lambda _{i}+\lambda _{j}}|\langle \psi _{i}|G| \psi _{j}\rangle |^{2},
\end{eqnarray*}

we have


(14) \begin{eqnarray*}
\mathcal {F}(\rho _{\rm f})\ge \lim _{\delta \rightarrow 0}4\frac{\Gamma (\rho _{\rm f}) -\mathcal {L}_\delta }{\delta ^2},
\end{eqnarray*}

where we used the fact that $\lambda _i+\lambda _j\le 1$, and $\Gamma (\cdot )$ denotes the purity of the state. For highly mixed states where the eigenvalues are almost degenerate, i.e. $\lambda _i\approx {1}/{d}$ for $1\le i\le d$, we have


(15) \begin{eqnarray*}
\mathcal {F}(\rho _{\rm f})\approx \lim _{\delta \rightarrow 0}2d\frac{\Gamma (\rho _{\rm f}) -\mathcal {L}_\delta }{\delta ^2}.
\end{eqnarray*}

### Experimental calibration of the precision of phase estimation

We can calibrate $\Delta \alpha _{\rm f}$ according to Equation (9), in which


\begin{eqnarray*}
\langle {\mathcal {O}}_{\rm rev}\rangle &=&{\rm Tr}({\rm e}^{-i\alpha G}\rho _{\rm f} {\rm e}^{i\alpha G}\mathcal {O}_{\rm rev}) \\
&=&\frac{1}{2^{N}}+\frac{\epsilon ^2}{2^{2N}}{\rm Tr}\left({\rm e}^{-i\alpha G}\rho _{\rm f}^\Delta {\rm e}^{i\alpha G}\rho _{\rm f}^\Delta \right), \\
\langle {\mathcal {O}}_{\rm rev}^2\rangle & =&{\rm Tr}\left({\rm e}^{-i\alpha G}\rho _{\rm f} {\rm e}^{i\alpha G}\mathcal {O}_{\rm rev}^2\right) \\
&=&\frac{1}{2^{2N}}+\frac{\epsilon ^2}{2^{2N+2}}\sum _i\gamma ^2_i \\
&& +\,\frac{\epsilon ^2}{2^{3N-1}}{\rm Tr}\big({\rm e}^{-i\alpha G}\rho _{\rm f}^\Delta {\rm e}^{i\alpha G}\rho _{\rm f}^\Delta \big)+O(\epsilon ^3).
\end{eqnarray*}

In experiment, following the method in [47,48], we extract $(\Delta \mathcal {O}_{\rm rev})^2=\langle \mathcal {O}_{\rm rev}^2\rangle -\langle \mathcal {O}_{\rm rev}\rangle ^2$ by substituting the experimental signal of ${\rm Tr}({\rm e}^{-i\alpha G}\rho _{\rm f}^\Delta {\rm e}^{i\alpha G}\rho _{\rm f}^\Delta )$ into the equations above with $N,\gamma _i,\epsilon$ being known. The derivation $d\langle \mathcal {O}_{\rm rev}\rangle /d\alpha$ is approximated with the finite-difference approach


(16) \begin{eqnarray*}
\frac{\langle \mathcal {O}_{\rm rev}\rangle }{d\alpha }\approx \frac{\langle \mathcal {O}_{\rm rev}\rangle _{\alpha +\delta ^\prime }-\langle \mathcal {O}_{\rm rev}\rangle _{\alpha -\delta ^\prime }}{2\delta ^\prime }
\end{eqnarray*}

with $\delta ^\prime ={\pi }/{50}$. For the experimental condition of $\epsilon \sim 10^{-5}$, we have the precision of the engineered state $\Delta \alpha _{\rm f}\sim 4.0\times 10^3$ at $\widetilde{\alpha }=0.08\pi$.

## Supplementary Material

nwaf091_Supplemental_File
