# Supplementary material for: Variational quantum metrology with the Loschmidt echo
Source: Natl Sci Rev. 2025 Mar 10;12(5):nwaf091. doi: 10.1093/nsr/nwaf091 (PMC12023863; doi:10.1093/nsr/nwaf091)
Supplement: nwaf091_Supplemental_File [file nwaf091_supplemental_file.pdf]

Variational Quantum Metrology with Loschmidt Echo: Supplemental Material

Ran Liu,[1,2,3,*] Ze Wu,[1,2,*] Xiaodong Yang,[3,4] Yuchen Li,[1,2] Hui Zhou,[5]
Zhaokai Li,[1,2,6] Yuquan Chen,[1,2] Haidong Yuan,[7,†] and Xinhua Peng[1,2,6,‡]

[1] *CAS Key Laboratory of Microscale Magnetic Resonance and School of Physical Sciences, University of Science and Technology of China, Hefei 230026, China*
[2] *CAS Center for Excellence in Quantum Information and Quantum Physics, University of Science and Technology of China, Hefei 230026, China*
[3] *Institute of Quantum Precision Measurement, State Key Laboratory of Radio Frequency Heterogeneous Integration, College of Physics and Optoelectronic Engineering, Shenzhen University, Shenzhen 518060, China*
[4] *Quantum Science Center of Guangdong-Hong Kong-Macao Greater Bay Area (Guangdong), Shenzhen 518045, China*
[5] *School of Physics, Hefei University of Technology, Hefei, Anhui 230009, China*
[6] *Hefei National Laboratory, University of Science and Technology of China, Hefei 230088, China*
[7] *Department of Mechanical and Automation Engineering, The Chinese University of Hong Kong, Shatin, Hong Kong SAR, China*

(Dated: February 13, 2025)

I. OPTIMAL QUENCH TIME UNDER ENCODING DYNAMICS

When extracting QFI from LE according to

$$\mathcal{F}\left[\rho_{\mathrm{f}}, G\right] \approx \lim_{\delta\to 0} 2d\frac{\Gamma(\rho_{\mathrm{f}}) - \mathcal{L}_\delta}{\delta^2}, \tag{S1}$$

there are three factors that can lead to the deviation from the theoretical QFI: 1) the eigenvalues of ρ_{f} are not exactly equal to $1/d$; 2) the higher-order (>2) terms are ignored in Eq. (S1) but they can be non-zero; 3) the experimental errors when measuring the LE. In the following, we give an analysis on how these factors affect the deviation and decide an optimal value of δ for the experimental extraction of the QFI.

1) In our experiments, the equilibrium state is $\rho_0 = (\mathbb{1} + \epsilon\rho_0^\Delta)/2^N$ with $\rho_0^\Delta = \gamma_{\mathrm{P}}\sigma_z^{\mathrm{P}} + \gamma_{\mathrm{H}}\sum_{j=1}^{9}\sigma_{j,z}^{\mathrm{H}}$. The eigenvalues can then be expressed as $\lambda_i = 1/2^N + \lambda_i^\Delta$ with $\lambda_i^\Delta \sim N\epsilon/2^N$. The error caused by taking λ_i as $1/d = 1/2^N$ can thus be obtained as

$$\begin{aligned}\mathcal{E}_1 =& 2\sum_{i,j}\left(\lambda_i-\lambda_j\right)^2 |\langle\psi_i|G|\psi_j\rangle|^2 \left|\frac{1}{\frac{2}{2^N}} - \frac{1}{\frac{2}{2^N}+\lambda_i^\Delta+\lambda_j^\Delta}\right| \\ =& 2\sum_{i,j}\left(\lambda_i^\Delta-\lambda_j^\Delta\right)^2 |\langle\psi_i|G|\psi_j\rangle|^2 \left|\frac{\lambda_i^\Delta+\lambda_j^\Delta}{\frac{2}{2^N}\left(\frac{2}{2^N}+\lambda_i^\Delta+\lambda_j^\Delta\right)}\right| \\ \sim& \frac{(N\epsilon)^3}{2^{N+2}}\end{aligned} \tag{S2}$$

2) For the Taylor series expansion of $\mathcal{L}_\delta$, its higher-order terms are non-negligible under finite δ. The leading term, i.e., the fourth-order term, is

$$\delta^4\left(\frac{1}{4}\sum_{i,j}\lambda_i\lambda_j|\langle\psi_i|G^2|\psi_j\rangle|^2 + \frac{1}{12}\sum_i\lambda_i^2\langle\psi_i|G^4|\psi_i\rangle - \frac{1}{3}\sum_{i,j}\lambda_i\lambda_j\mathrm{real}\left(\langle\psi_j|G|\psi_i\rangle\langle\psi_i|G^3|\psi_j\rangle\right)\right). \tag{S3}$$

Consequently, it leads to the error on QFI

$$\mathcal{E}_2 \sim \frac{\delta^2}{2^{N-1}}. \tag{S4}$$

---

*These authors contribute equally
†Electronic address: hdyuan@mae.cuhk.edu.hk
‡Electronic address: xhpeng@ustc.edu.cn

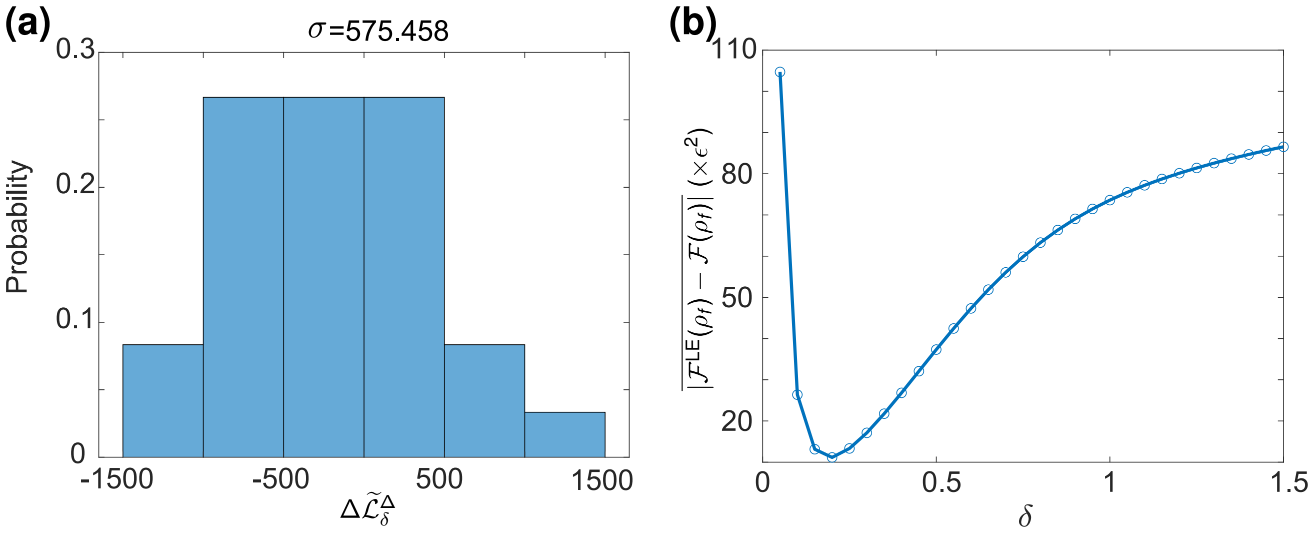

FIG. S1: (a) Calibration of experimental error for measuring $\mathcal{L}_\delta^\Delta$. (b) Numerical simulation of deviations on QFI under different quench time δ. When $\delta = 0.2$, the error gets its minimum.

3) As we mentioned in the main text, $\mathcal{L}_\delta$ is experimentally obtained by measuring its deviation part $\mathcal{L}_\delta^\Delta$, i.e., $\mathcal{L}_\delta = 1/2^N + \epsilon^2 \mathcal{L}_\delta^\Delta$. The deviations of the experimental results $\mathcal{L}_\delta^{\Delta,\rm exp}$ from the theoretical one leads to the error on QFI,

$$\mathcal{E}_3 \sim \frac{2^{N+1}\epsilon^2 \Delta\mathcal{L}_\delta^\Delta}{\delta^2} \tag{S5}$$

with $\Delta\mathcal{L}_\delta^\Delta = \mathcal{L}_\delta^{\Delta,\rm exp} - \mathcal{L}_\delta^{\Delta,\rm theo}$. We calibrate the deviations from experiments by measuring $\mathcal{L}_\delta^{\Delta,\rm exp}$ under 80 different $U_{\rm E}(\boldsymbol{\theta})$ and calculating their standard deviation as $\sigma = 575.46$. The probability histogram is shown in Fig. S1 (a).

Since $\epsilon \sim 10^{-5}$ at the thermal polarization, $\mathcal{E}_1$ is much smaller than $\mathcal{E}_2$ and $\mathcal{E}_3$, thus negligible. The influences of δ on $\mathcal{E}_2$ and $\mathcal{E}_3$ are opposite, as Eq.(S4) shows $\mathcal{E}_2$ increases with δ while Eq.(S5) shows $\mathcal{E}_3$ decreases with δ. Thus there exists a tradeoff and an optimal δ needs to be determined to minimize the total error. As shown in Fig. S1 (b), we numerically simulate the mean of the difference between the QFI obtained from the LE under experimental errors and the theoretical one, i.e., $\overline{|\mathcal{F}^{\rm LE}(\rho_{\rm f}) - \mathcal{F}(\rho_{\rm f})|}$, under 50 different $U_{\rm E}(\boldsymbol{\theta})$ for each δ. The experimental error is simulated by adding artificial fluctuations on $\mathcal{L}_\delta^\Delta$ obeying normal distribution with a standard deviation σ. From the simulation we can see that the error is minimal at $\delta = 0.2$, which is taken as the experimental quench time.

II. DETAILS OF THE NELDER-MEAD ALGORITHM

The Nelder-Mead algorithm provides a useful procedure for searching the minimum of a given function without derivatives. By rescaling a simplex consisting of $n+1$ vertices iteratively, this algorithm attempts to repalce the worst vertex by a better one. Here each vertex represents a sequence of n parameters that can be tuned in the parametrized quantum circuit(PQC). The procedure of the Nelder-Mead algorithm is described as below.

1. **Ording**: Calculate the cost function of $n+1$ initial vertices and sort them as

$$f(\boldsymbol{\theta}^{(1)}) \le f(\boldsymbol{\theta}^{(2)}) \le \cdots \le f(\boldsymbol{\theta}^{(n+1)}). \tag{S6}$$

where the cost function is LE with $f(\boldsymbol{\theta}^{(i)}) = \mathcal{L}_\delta(\boldsymbol{\theta}^{(i)})$. For the 3-layer PQC used in the experiment we have $n = 6$.

2. **Centroid**: Evaluate the cost function of the centroid of the best n points, $f(\boldsymbol{\theta}^{\rm ave})$, here $\boldsymbol{\theta}^{\rm ave} = \sum_{i=1}^n \boldsymbol{\theta}^{(i)}/n$.

3. **transformation**: Replace the worst vertex $\boldsymbol{\theta}^{(n+1)}$ and the corresponding cost function with a better one by using reflection, expansion, contraction or shrink. The concrete rules for this transformation are as follows:

(1) **Reflect**: Calculate the cost function of $f_r = f(\boldsymbol{\theta}^r)$ with $\boldsymbol{\theta}^r := \boldsymbol{\theta}^{\text{ave}} + \alpha(\boldsymbol{\theta}^{(n+1)} - \boldsymbol{\theta}^{(n)})$ as the reflection point. α is the reflection coefficient and set as 1. If $f_1 \le f_r \le f_n$, accept $\boldsymbol{\theta}^r$.

(2) **Expand**: If $f_r < f_1$, calculate $f_e = f(\boldsymbol{\theta}^e)$ with $\boldsymbol{\theta}^e := \boldsymbol{\theta}^{\text{ave}} + \gamma \cdot \alpha(\boldsymbol{\theta}^{(n+1)} - \boldsymbol{\theta}^{(n)})$ as the expansion point and γ is the expansion coefficient and set as 2. If $f_e < f_r$ accept $\boldsymbol{\theta}^e$. Otherwise, accept $\boldsymbol{\theta}^r$.

(3) **Contract**: (3a) If $f_n \le f_r \le f_{n+1}$, calculate $f_c := f(\boldsymbol{\theta}^c)$ with $\boldsymbol{\theta}^c := \boldsymbol{\theta}^{\text{ave}} + \beta \cdot \alpha(\boldsymbol{\theta}^{(n+1)} - \boldsymbol{\theta}^{(n)})$ as the outside contraction point and β is the contraction coefficient and set as 0.5. If $f_c \le f_r$, accept f_c. Otherwise, perform a shrink transformation. (3b) If $f_r \ge f_{n+1}$, calclulate $f_c := f(\boldsymbol{\theta}^c)$ with $\boldsymbol{\theta}^c := \boldsymbol{\theta}^{\text{ave}} - \beta \cdot \alpha(\boldsymbol{\theta}^{(n+1)} - \boldsymbol{\theta}^{(n)})$ as the inside contraction point and β is set as 0.5. If $f_c \le f_r$, accept f_c. Otherwise, perform a shrink transformation.

(4) **Shrink**: Calculate $f_i := f(\boldsymbol{\theta}^{(i)})$ with $\boldsymbol{\theta}^{(i)} := \boldsymbol{\theta}^{(1)} + (1-\delta)\boldsymbol{\theta}^{(i)}$ and $i = 2, 3, ..., n+1$. δ is the shrinkage coefficient and set as 0.5.

4. **Termination tests**: If the result satisfies the stopping condition, terminate the iterations. Otherwise, change the iteration number as $l = l + 1$ and continue at **Ording**.

Here the stopping condition is whether the optimized QFI is close to the optimum. We simulate 30 rounds of experimental iterations, and in each round the initial vertices ($l = 1$) is generated randomly with $\boldsymbol{\theta}_i \in [0, 2\pi]$ and $i = 1, 2, ..., n+1$. To take the experimental errors into account, we add fluctuations on theoretical $\mathcal{L}_\delta^\Delta$ with the standard deviation σ as obtained in Sec. I. The simulated result in Fig. S2 (a) shows that the optimization converges and approaches to its optimum when $l \ge 70$. Consequently, we stop the experimental iterations when $l = 70$.

To improve the efficiency of the optimization, we further modify the initial simplex ($l = 1$) as [1]

$$\theta_j^{(i)} = \begin{cases} \theta_j^{(1)} + 2\pi(\sqrt{n+1} - 1) & i \neq j+1 \&\& i > 1 \\ \theta_j^{(1)} + 2\pi(\sqrt{n+1} + n - 1) & i = j+1 \&\& i > 1 \end{cases} \tag{S7}$$

and $\boldsymbol{\theta}^{(1)} = (0, 0, ...0)^{\text{T}}$. We simulate 100 rounds of optimization under this initial simplex and stop the iteration when $l = 70$. The statistical result of optimized QFI is show in Fig. S2 (b). Compared with the case of randomly generated initial simplex, as shown in Fig. S2 (c)-(k), the modified one shows higher expectation to approach a large QFI under noise, thus adopted in the experiment.

III. PRECISION BOUND OF MIXED-STATE QUANTUM METROLOGY

We study the achievable precision using initially mixed probe states, where the initial state can be transformed with unitary operations which does not alter its spectrum.

For a quantum system initialized as a pure state for the estimation of θ in the dynamics $e^{-i\theta H}$, the optimal probe state is given by $|\psi_{\text{Opt}}\rangle = (|h_{\max} + e^{i\varphi}|h_{\min})/\sqrt{2}$, where $|h_{\max,\min}\rangle$ are the eigenvectors of H corresponding to the maximal and minimal eigenvalues, and φ is an arbitrary phase. In the case of $H = \sum_i \sigma_z^i/2$, $|\psi_{\text{Opt}}\rangle$ corresponds to the NOON state and exhibits a $10\lg(N)$ dB enhancement in precision over SQL. However, for mixed initial state the situation is more complicated. For a d-dimensional mixed initial state, $\rho = \sum_{k=1}^d p_k |\psi_k\rangle\langle\psi_k|$, to estimate θ in the dynamics $e^{-i\theta H}$ with $H = \sum_{k=1}^d h_k |h_k\rangle\langle h_k|$, where $p_1 \ge \cdots \ge p_d$ and $h_1 \ge \cdots \ge h_d$ are ordered, the optimal probe state that can be obtained from the initial probe state under unitary operation is given by[2]

$$\rho_{\text{Opt}} = \sum_{k=1}^d p_k |\phi_k\rangle\langle\phi_k| \tag{S8}$$

with

$$|\phi_k\rangle = \begin{cases} \frac{|h_k\rangle + |h_{d-k+1}\rangle}{\sqrt{2}} & \text{if } 2k < d+1 \\ |h_k\rangle & \text{if } 2k = d+1 \\ \frac{|h_k\rangle - |h_{d-k+1}\rangle}{\sqrt{2}} & \text{if } 2k > d+1. \end{cases} \tag{S9}$$

The corresponding Quantum Fisher Information (QFI) is given by,

$$\mathcal{F}_{\text{Opt}} = \frac{1}{2}\sum_{k=1}^d p_{k,d-k+1}(h_k - h_{d-k+1})^2 \tag{S10}$$

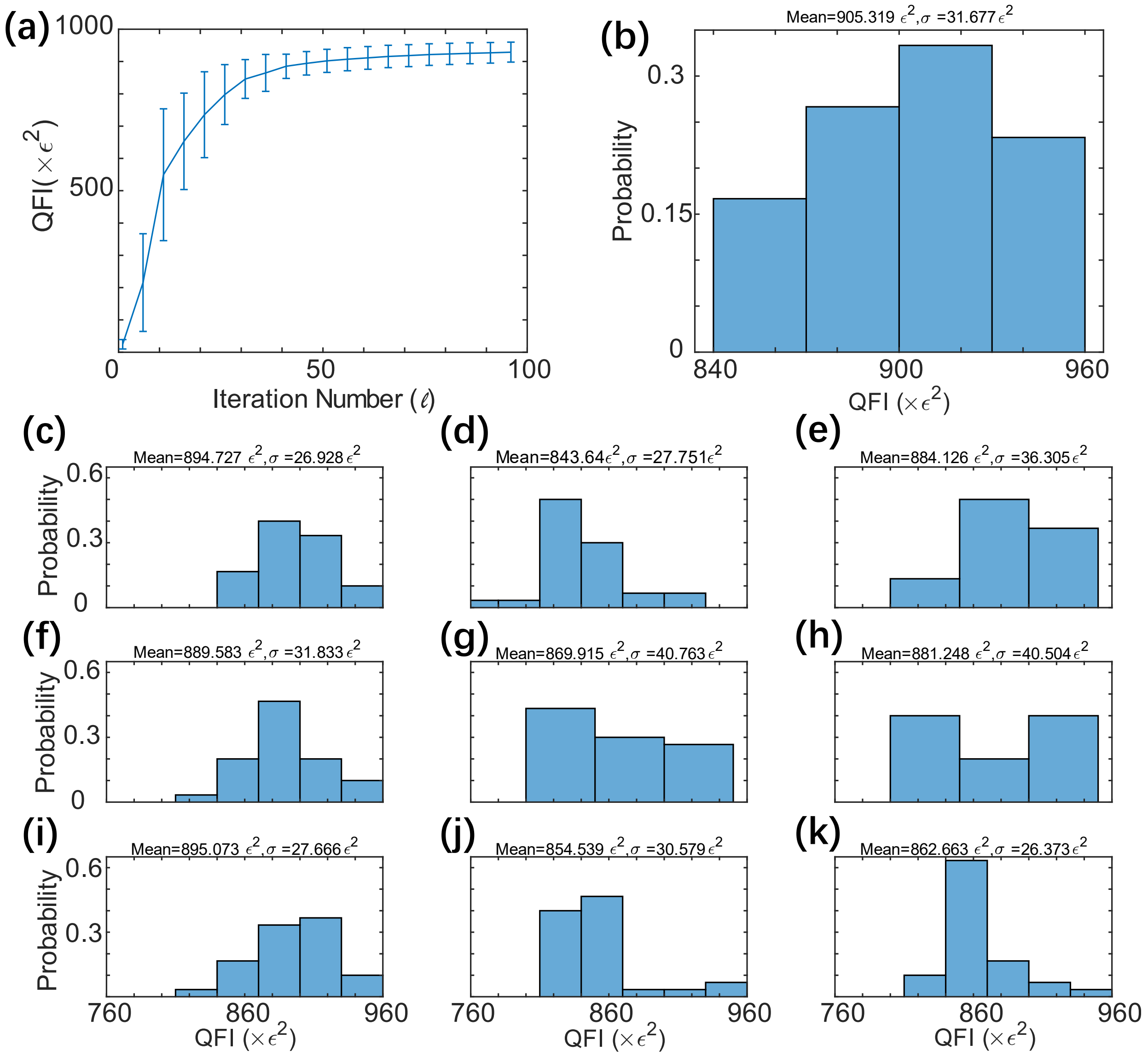

FIG. S2: Simulated iterations with NM algorithm. (a) Statistical result of 30 rounds of iteration under different initial simplexes and random fluctuation. When $l \geq 70$, the optimization converges and approaches to its optimum. Statistical result of optimized QFI obtained from 100 rounds of iteration with (b) modified initial simplex and (c-k) random initial simplex. The modified one shows higher expectation to approach a large QFI.

with

$$p_{k,l} = \begin{cases} 0 & \text{if } p_k = p_l = 0 \\ \frac{(p_k - p_l)^2}{p_k + p_l} & \text{else.} \end{cases} \tag{S11}$$

To investigate the scaling of the precision bound for mixed states, we consider a specific N-qubit mixed-state system initialized as $\rho = \rho_i^{\otimes N}$, where each qubit is given by $\rho_i = (\mathbb{1} + \epsilon\sigma_z)/2$, and $\epsilon \in [0, 1]$ represents polarization. This state is similar to a thermal state in NMR experiments when $\epsilon \sim 10^{-5}$. Under the encoding dynamics $H = \sum_i \sigma_z^i/2$, the classical uncorrelated probe state generated by optimal local unitary operations on individual spin is $\rho_{i,x}^{\otimes N}$, where $\rho_{i,x} = (\mathbb{1} + \epsilon\sigma_x)/2$. The corresponding QFI of $\rho_{i,x}^{\otimes N}$ is $\mathcal{F}_{\rm cl} = \epsilon^2 N$, thus a scaling of standard quantum limit (SQL) [2]. The QFI of the probe state generated by the optimal global (entangling) unitary operation is given by

$$\mathcal{F}_{\rm Opt} = \sum_{m=0}^{N-1} \frac{(N-2m)^2 \left[(1+\epsilon)^{N-m}(1-\epsilon)^m - (1+\epsilon)^m(1-\epsilon)^{N-m}\right]^2}{2^N \left[(1+\epsilon)^{N-m}(1-\epsilon)^m + (1+\epsilon)^m(1-\epsilon)^{N-m}\right]} \begin{pmatrix} N-1 \\ m \end{pmatrix} \geq \epsilon^2 N^2. \tag{S12}$$

The enhancement over the $F_{\rm cl}$ of classical uncorrelated state is thus greater than N.

In our experiment, the initial state of each spin is $\rho_i' = (\mathbb{1} + \epsilon\gamma_i\sigma_z)/2$, where γ_i related to the gyromagnetic ratios of different nuclei. This is slightly different from the state $\rho_i^{\otimes N}$ with $\rho_i = (\mathbb{1} + \epsilon\sigma_z)/2$ as we mentioned above, which is due to different gyromagnetic ratios in the molecule used in our experiment. For the experimental thermal polarization $\epsilon \sim 10^{-5}$, we have $\bigotimes_{i=1}^N \rho_i' \approx (\mathbb{1} + \epsilon\rho_{\rm eq}^{\Delta})/2^N$ with $\rho_{\rm eq}^{\Delta} = \sum_{j=1}^N \gamma_j\sigma_z^j$, i.e., the equilibrium state in our manuscript. Similarly, the classical uncorrelated state that can be generated from $\bigotimes_{i=1}^N \rho_i'$ via optimal local unitary operation on individual spin is $\bigotimes_{i=1}^N \rho_{i,x}'$ with $\rho_{i,x}' = (\mathbb{1} + \epsilon\gamma_i\sigma_x)/2$, whose QFI equals to $\epsilon^2\sum_i^N \gamma_i^2$. For the 10-spin TMP molecule in our experiment, we have $\gamma_1 = 0.8, \gamma_{2,3,...10} = 2$, this gives the maximal QFI under the local unitary operation as $36\epsilon^2$, which serves as the classical precision bound for $N = 10$. The QFI of the optimal state, which can be obtained from Eq. (S10), is $989\epsilon^2$, a 14.4 dB improvement over the classical case. This is different from maximal 10 dB improvement in the case of pure initial probe state.

IV. STRUCTURE OF VARIATIONAL QUANTUM CIRCUIT AND ITS REACHABLE SET

The structure of variational quantum circuit (VQC) used to realize the engineering operation $U_{\rm E}$ is shown in Fig. S3. The PQC has 3 layers with the first layer being local rotations along y-axis while the others consisting of an entangling gate and local rotations. The entangling gate is realized by the free evolution under the system Hamiltonian $H_{\rm NMR} = \frac{\pi}{2}J_{\rm PH}\sigma_z^1 \otimes \sum_{j=2}^{10}\sigma_z^j$ with the interval $\tau = 1/2J_{\rm PH}$, and the parameters are the angles of local rotation $\boldsymbol{\theta} := (\theta_1, \theta_2, ...\theta_6)^{\rm T}$. We now show that the reachable set of quantum state generated by $U_{\rm E}(\boldsymbol{\theta})$, denote as $\mathcal{R}_{U_{\rm E}}$, includes the optimal probe state.

The polarized part of initial equilibrium state can be written as $\gamma_{\rm P}Z(I^{\otimes 9})/9 + \gamma_{\rm H}I(ZI^{\otimes 8})$, here X, Y, Z, I represent $\sigma_{x,y,z}, \mathbb{1}_2$ respectively, and $(\cdot)$ represent the summation over all the indistinguishable permutations, such as $(I^{\otimes 9}) := \sum_{i=1}^9 I^{\otimes 9}\cdots, (ZI^{\otimes 8}) := ZII\cdots + IZI\cdots + IIZ\cdots + ...$. The evolved state after $e^{-i\left(\theta_1\sigma_y/2 + \theta_4\sum_{j=2}^{10}\sigma_y^z/2\right)}$, corresponding to stage ① in Fig. S3, is

$$\begin{aligned}
①: &Z(I^{\otimes 9}) \to \cos\theta_1 Z(I^{\otimes 9}) + \sin\theta_1 X(I^{\otimes 9}) \\
&I(ZI^{\otimes 8}) \to \cos\theta_4 I(ZI^{\otimes 8}) + \sin\theta_4 I(XI^{\otimes 8}) \\
&\rho_{①} = \gamma_{\rm P}\left[\cos\theta_1 Z(I^{\otimes 9}) + \sin\theta_1 X(I^{\otimes 9})\right]/9 + \gamma_{\rm H}\left[\cos\theta_4 I(ZI^{\otimes 8}) + \sin\theta_4 I(XI^{\otimes 8})\right]
\end{aligned} \tag{S13}$$

Since $\left[\sigma_z^1\sigma_z^i, \sigma_z^1\sigma_z^j\right] = 0$ for $i, j = 1, 2, ...9$, the different terms in $H_{\rm NMR}$ can then be applied in turn,

$$\begin{aligned}
②: &Z(I^{\otimes 9}) \to Z(I^{\otimes 9}) \\
&X(I^{\otimes 9}) \xrightarrow{I_z^0 I_z^1} 9YZI^{\otimes 8} \xrightarrow{I_z^0 I_z^2} -9XZZI^{\otimes 7} \xrightarrow{I_z^0 I_z^3} \cdots \xrightarrow{I_z^0 I_z^9} Y(Z^{\otimes 9}) \\
&I(ZI^{\otimes 8}) \to I(ZI^{\otimes 8}) \\
&I(XI^{\otimes 8}) \to Z(YI^{\otimes 8}) \\
&\rho_{②} = \gamma_{\rm P}\left[\cos\theta_1 Z(I^{\otimes 9}) + \sin\theta_1 Y(Z^{\otimes 9})\right]/9 + \gamma_{\rm H}\left[\cos\theta_4 I(ZI^{\otimes 8}) + \sin\theta_4 Z(YI^{\otimes 8})\right].
\end{aligned} \tag{S14}$$

Similarly, we have

$$\begin{aligned}
③: &Z(I^{\otimes 9}) \to \cos\theta_2 Z(I^{\otimes 9}) + \sin\theta_2 X(I^{\otimes 9}) \\
&Y(Z^{\otimes 9}) \to \sum_{i=1}^{10}\cos^{10-i}\theta_5\sin^{i-1}\theta_5 Y(Z^{\otimes 10-i}X^{\otimes i-1}) \\
&I(ZI^{\otimes 8}) \to \cos\theta_5 I(ZI^{\otimes 8}) + \sin\theta_5 I(XI^{\otimes 8}) \\
&Z(YI^{\otimes 8}) \to \cos\theta_2 Z(YI^{\otimes 8}) + \sin\theta_2 X(YI^{\otimes 8}) \\
&\rho_{③} = \gamma_{\rm P}\left[\cos\theta_1(\cos\theta_2 Z(I^{\otimes 9}) + \sin\theta_2 X(I^{\otimes 9})) + \sin\theta_1(\sum_{i=1}^{10}\cos^{10-i}\theta_5\sin^{i-1}\theta_5 Y(Z^{\otimes 10-i}X^{\otimes i-1}))\right]/9 \\
&+ \gamma_{\rm H}\left[\cos\theta_4(\cos\theta_5 I(ZI^{\otimes 8}) + \sin\theta_5 I(XI^{\otimes 8})) + \sin\theta_4(\cos\theta_2 Z(YI^{\otimes 8}) + \sin\theta_2 X(YI^{\otimes 8}))\right]
\end{aligned} \tag{S15}$$

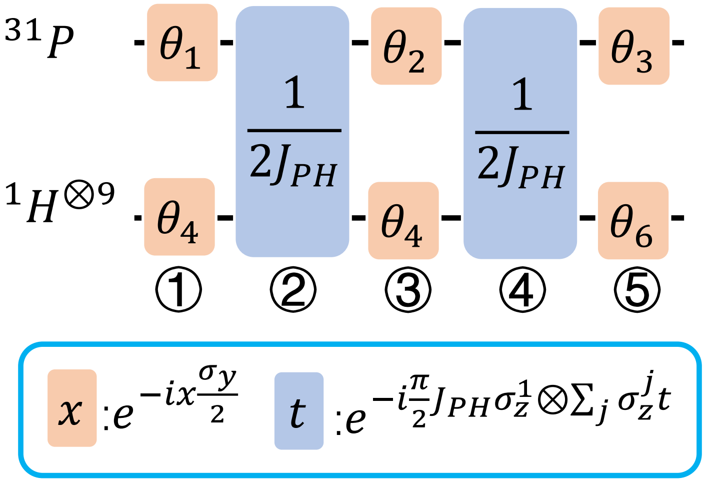

FIG. S3: The structure of PQC used to engineer quantum probe, which consists of single-qubit rotations and entangling gates. Its reachable set of quantum states can cover the optimal quantum probe when $\sin\theta_2 = 1, \sin\theta_6 = 1, \cos\theta_1\cos\theta_3\sin\theta_4 = 1$.

$$
\begin{aligned}
④ :& Z(I^{\otimes 9}) \to Z(I^{\otimes 9}) \\
& X(I^{\otimes 9}) \to Y(Z^{\otimes 9}) \\
& Y(Z^{\otimes 9}) \xrightarrow{I_z^1 I_z^2} -9XIZ^{\otimes 8} \xrightarrow{I_z^1 I_z^3} -9YIIZ^{\otimes 7} \xrightarrow{I_z^1 I_z^4} \dots \xrightarrow{I_z^1 I_z^{10}} X(I^{\otimes 9}) \\
& Y(XZ^{\otimes 8}) \to Y(XI^{\otimes 8}) \\
& \text{due to } YXZ^{\otimes 8} \xrightarrow{I_z^1 I_z^2} YXZ^{\otimes 8} \xrightarrow{I_z^1 I_z^3} XXIZ^{\otimes 7} \xrightarrow{I_z^1 I_z^4} \dots \xrightarrow{I_z^1 I_z^{10}} YXI^{\otimes 8} \\
& \dots \\
& Y(ZX^{\otimes 8}) \to X(IX^{\otimes 8}) \\
& \text{due to } YZX^{\otimes 8} \xrightarrow{I_z^1 I_z^2} XIX^{\otimes 8} \xrightarrow{I_z^1 I_z^3} XIX^{\otimes 8} \xrightarrow{I_z^1 I_z^4} \dots \xrightarrow{I_z^1 I_z^{10}} XIX^{\otimes 8} \\
& Y(X^{\otimes 9}) \to Y(X^{\otimes 9}) \\
& I(ZI^{\otimes 8}) \to I(ZI^{\otimes 8}) \\
& I(XI^{\otimes 8}) \to Z(YI^{\otimes 8}) \\
& Z(YI^{\otimes 8}) \to I(XI^{\otimes 8}) \\
& X(YI^{\otimes 8}) \to X(YZ^{\otimes 8}) \\
& \text{due to } XYI^{\otimes 8} \xrightarrow{I_z^1 I_z^2} XYI^{\otimes 8} \xrightarrow{I_z^1 I_z^3} YYZI^{\otimes 7} \xrightarrow{I_z^1 I_z^4} \dots \xrightarrow{I_z^1 I_z^{10}} XYZ^{\otimes 8}
\end{aligned}
\tag{S16}
$$

$$
\begin{aligned}
\rho_{④} = {} & \gamma_{\mathrm{P}} \left[\cos\theta_1 (\cos\theta_2 Z(I^{\otimes 9}) + \sin\theta_2 Y(Z^{\otimes 9})) + \sin\theta_1 \sum_{i=1}^{10} \cos^{10-i}\theta_5 \sin^{i-1}\theta_5 (X \mathrm{or} Y)(I^{\otimes 10-i} X^{\otimes i-1}) \right] /9 \\
& + \gamma_{\mathrm{H}} \left[\cos\theta_4 (\cos\theta_5 I(ZI^{\otimes 8}) + \sin\theta_5 Z(YI^{\otimes 8})) + \sin\theta_4 (\cos\theta_2 I(XI^{\otimes 8}) + \sin\theta_2 X(YZ^{\otimes 8})) \right]
\end{aligned}
\tag{S17}
$$

$$
\begin{aligned}
&⑤ : Z(I^{\otimes 9}) \to \cos\theta_3 Z(I^{\otimes 9}) + \sin\theta_3 X(I^{\otimes 9}) \\
&Y(Z^{\otimes 9}) \to \sum_{i=1}^{10} \cos^{10-i}\theta_6 \sin^{i-1}\theta_6 Y(Z^{\otimes 10-i} X^{\otimes i-1}) \\
&X(I^{\otimes 9}) \to \cos\theta_3 X(I^{\otimes 9}) - \sin\theta_3 Z(I^{\otimes 9}) \\
&Y(XI^{\otimes 8}) \to \cos\theta_6 Y(XI^{\otimes 9}) - \sin\theta_6 Y(ZI^{\otimes 9}) \\
&\cdots \\
&X(IX^{\otimes 8}) \xrightarrow{\theta_3 I_y^1} \cos\theta_3 X(IX^{\otimes 8}) - \sin\theta_3 Z(IX^{\otimes 8}) \\
&\xrightarrow{\theta_6 \sum_i I_y^i} \cos\theta_3 (\sum_{i=1}^{9} \cos^{9-i}\sin^{i-1}\theta_6 X(IX^{\otimes 9-i} Z^{\otimes i-1})) - \sin\theta_3 (\sum_{i=1}^{9} \cos^{9-i}\sin^{i-1}\theta_6 Z(IX^{\otimes 9-i} Z^{\otimes i-1})) \\
&Y(X^{\otimes 9}) \to \sum_{i=1}^{10} \cos^{10-i}\theta_6 (-\sin\theta_6)^{i-1} Y(X^{\otimes 10-i} Z^{\otimes i-1}) \\
&I(ZI^{\otimes 8}) \to \cos\theta_6 I(ZI^{\otimes 8}) + \sin\theta_6 I(XI^{\otimes 8}) \\
&Z(YI^{\otimes 8}) \to \cos\theta_3 Z(YI^{\otimes 8}) + \sin\theta_3 X(YI^{\otimes 8}) \\
&I(XI^{\otimes 8}) \to \cos\theta_6 I(XI^{\otimes 8}) - \sin\theta_6 I(ZI^{\otimes 8}) \\
&X(YZ^{\otimes 8}) \xrightarrow{\theta_3 I_y^1} \cos\theta_3 X(YZ^{\otimes 8}) - \sin\theta_3 Z(YZ^{\otimes 8}) \\
&\xrightarrow{\theta_6 \sum_i I_y^i} \cos\theta_3 (\sum_{i=1}^{9} \cos^{9-i}\sin^{i-1}\theta_6 X(YZ^{\otimes 9-i} X^{\otimes i-1})) - \sin\theta_3 (\sum_{i=1}^{9} \cos^{9-i}\sin^{i-1}\theta_6 Z(YZ^{\otimes 9-i} X^{\otimes i-1}))
\end{aligned}
\tag{S18}
$$

Consequently, $\mathcal{R}_{U_\mathrm{E}}$ can be given by ⑤ with $\theta_1, \theta_2, ...\theta_6 \in [0, 2\pi]$.

The form of optimal probe is given by Eq. (S8), and it guarantees that the amplitude of highest-order coherence is maximal, as the elements with higher-order coherence in probe state show faster phase accumulation. Under the encoding dynamics $G = \sum_{k=1}^{10} \sigma_z/2$, X, Y contribute to the order of coherence while I, Z do not. In the following, we consider the highest-order, i.e., 10-order, coherence in probe,

$$
\rho^{\Delta}_{(10)} = \frac{\gamma_\mathrm{P}}{9}\left[\cos\theta_1 \sin\theta_2 \sin^9\theta_6 Y(X^{\otimes 9}) + \sin\theta_1 \sin^9\theta_5 \cos^9\theta_6 Y(X^{\otimes 9})\right] + \gamma_\mathrm{H} \sin\theta_4 \sin\theta_2 \cos\theta_3 \sin^8\theta_6 X(YX^{\otimes 8}). \tag{S19}
$$

When $\sin\theta_2 = 1, \sin\theta_6 = 1, \cos\theta_1\cos\theta_3\sin\theta_4 = 1$, the contriubtion of $\rho^{\Delta}_{(10)}$ is maximized and the corresponding probe is optimal. So $\mathcal{R}_{U_\mathrm{E}}$ can cover the optimal probe.

V. EXPERIMENTAL PROCEDURE FOR MEASURING LE AND ITS SCALABILITY IN QUANTUM CIRCUITS

The LE can be effectively constructed in the NMR experiments by performing a forward evolution U_E, a perturbation $e^{-i\delta G}$ with $\delta \to 0$, and a reverse evolution $U_E^\dagger$ on the quantum system, and finally extracted by measuring the expectation of σ_z of each spin. Specifically, the LE can be expressed as $\mathcal{L}_\delta = \mathrm{Tr}\left[V_\delta(\boldsymbol{\theta})\rho_0 V_\delta^\dagger(\boldsymbol{\theta})\rho_0\right]$, where $V_\delta(\boldsymbol{\theta}) = U_\mathrm{E}^\dagger(\boldsymbol{\theta}) e^{-i\delta G} U_\mathrm{E}(\boldsymbol{\theta})$, and it can be experimentally extracted via the following procedures in the NMR experiments, as shown in Fig. S4.

1). Start from the equilibrium state at room temperature of the NMR system $\rho_\mathrm{eq} = (\mathbb{1} + \epsilon\rho^{\Delta}_\mathrm{eq})/2^N$. Here, $\rho^{\Delta}_\mathrm{eq} = \sum_{j=1}^N \gamma_j \sigma_z^j/2$, ϵ is the thermal polarization ($\sim 10^{-5}$) and γ_j is the relative gyromagnetic ratio of the corresponding nuclear.

2). Perform a forward evolution U_E, which consists of single-qubit rotations and free evolution under the system Hamiltonian $H_\mathrm{NMR} = \frac{\pi}{2} J_\mathrm{PH} \sigma_z^1 \otimes \sum_{j=2}^{10} \sigma_z^j$ with a duration $\tau = 1/2J_\mathrm{PH}$.

3). Perform the perturbation $e^{-i\delta G}$ with $G = \sum_{j=1}^N \sigma_z^j$. This is realized by single-qubit rotations with a small angle 2δ.

4). Perform the reverse evolution $U_\mathrm{E}^\dagger$ by substituting each operation in U_E with its inverse and applying them in reverse order. For single-qubit rotations, the reverse is achieved by adjusting the phase of the rotation. For free evolution under H_NMR, the reverse is implemented by applying π pulses to the first spin at both the beginning and the end of the free evolution.

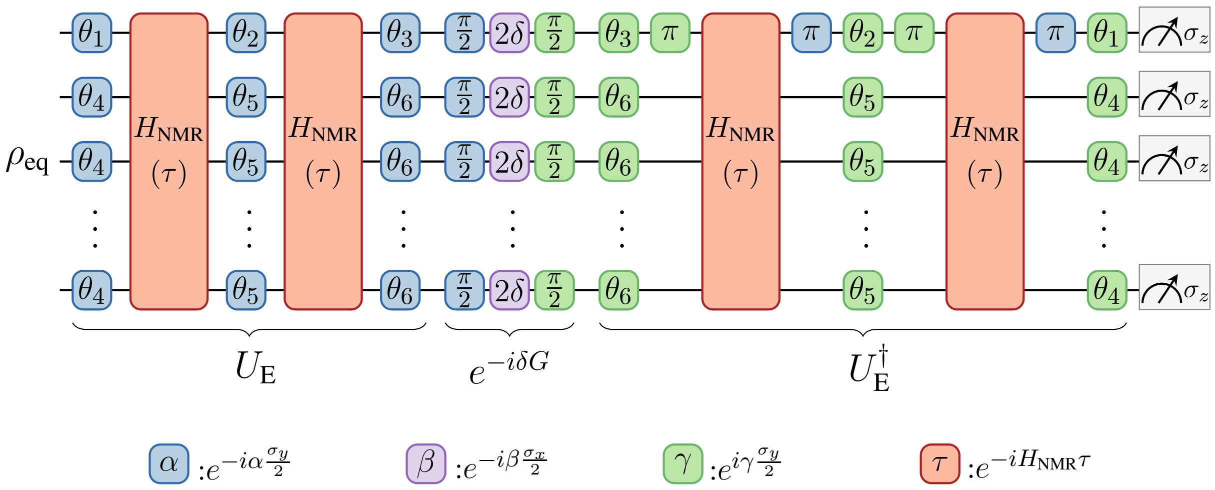

FIG. S4: Quantum circuit for measuring LE in the NMR experiment.

5). Project the evolved state onto the initial state ρ_{eq}. For the initial equilibrium state, this process can be equally expressed as $\mathcal{L}_\delta = c_0 + \sum_{j=1}^{N} c_j \text{Tr}\left[V_\delta(\boldsymbol{\theta})\rho_0 V_\delta^\dagger(\boldsymbol{\theta})\sigma_z^j\right]$ with c_j being constants. So in the NMR experiment we measure $\text{Tr}\left[V_\delta(\boldsymbol{\theta})\rho_0 V_\delta^\dagger(\boldsymbol{\theta})\sigma_z^j\right]$, i.e., the z-direction polarizations of each spin. Obviously, the measurement overhead scales linearly with the size of our system.

Apart from the implementation in the 10-spin NMR experiment, we continue to analyze the scalability of measuring LE when N is large and its feasibility in other quantum systems. Here, we employed the hardware-efficient Ansätze for the design of variational quantum circuits U_{E}. These circuits are constructed from entangling layers W_l and parameterized single-qubit rotation layers $U_l(\boldsymbol{\theta}_l)$, expressed as:

$$U_E = \prod_{l=1}^{L} U_l\left(\boldsymbol{\theta}_l\right) W_l, \tag{S20}$$

where L denotes the number of layers. Moreover, these circuits employ a limited set of quantum gates, i.e., typically two-qubit entangling gates for W_l and single-qubit gates for $U_l(\boldsymbol{\theta}_l)$. This ensures the efficient implementation of the inverse of each gate in the circuit. According to

$$U_E^\dagger = \prod_{l=1}^{L} W_{L-l+1}^\dagger U_{L-l+1}^\dagger\left(\boldsymbol{\theta}_{L-l+1}\right), \tag{S21}$$

we can implement the reverse $U_E^\dagger$ by applying the reverse of each gate in U_E in the reverse order. This ensures that the resource requirements for realizing $U_E^\dagger$ remain comparable to those for U_E, making the approach feasible when the size of system grows.

This Ansatz also accommodates constraints such as limited qubit connectivity and restricted gate sets of quantum hardware, thus can be implemented on other quantum systems. In our experiment, the entangling gate is $e^{-i\pi\sigma_z^1\sigma_z^i}, (i > 1)$ and can be reversed using π-pulses. This design can be extended to other physical systems with scalable two-qubit gates, such as CNOT or CZ gates for superconducting qubits or XX gates for trapped ions. For example, the quantum circuit for measuring LE with the Hardware-efficient Ansatz containing CNOT gates can be implemented in Fig. S5.

VI. ANALYSIS OF EXPERIMENTAL ERRORS AND TECHNIQUES FOR MITIGATING THEM

As mentioned in the main text, the relative error of measured $\mathcal{L}_\delta^\Delta$ during the iteration is 1.35%. The sources of experimental errors include the pulse error, relaxation and measurement error. We employ different techniques to mitigate them. A detailed introduction of these techniques and the analysis of the experimental errors, aided with numerical simulations, are listed in the following.

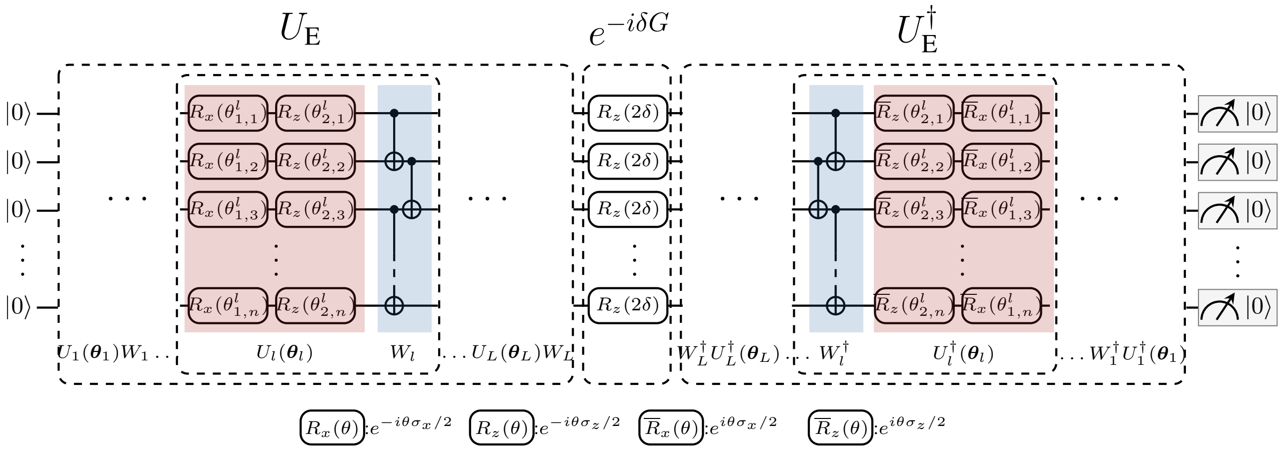

FIG. S5: Quantum circuit for measuring LE with the Hardware-efficient Ansatz containing CNOT gates.

1. **Pulse error**: The pulse error can lead to the deviation between the actually implemented rotation angles and the desired ones. This is mainly caused by the imperfect calibration of $\pi/2-$pulse in NMR experiment. To improve the robustness of the pulses, we replace the regular pulses with the BB1 sequence $R_\phi(\theta) \to R_\phi(\pi)R_{3\phi}(2\pi)R_\phi(\pi)R_\phi(\theta)$, where θ and ϕ is the target angle and phase, $\phi = \arccos(-\theta/4\pi)$. We simulate the pulse error by adding random fluctuation with 5% relative distortions to the amplitude of the pulses. The result shows that the relative error of $\mathcal{L}_\delta^\Delta$ caused by the pulse error decreases from 8.1% to 4.5×10^{-2}% after applying the BB1 sequence. Thus the pulse error is significantly suppressed.

2. **Relaxation**: The quantum probe inevitably interacts with the environment during the evolution. The noise in NMR experiment can be described by the phase damping channel, $\mathcal{E}_{\mathrm{PD}}$, and the generalized amplitude damping channel, $\mathcal{E}_{\mathrm{GAD}}$. The effect of the phase damping channel on the density matrix ρ can be approximately expressed as $\rho \to \mathcal{E}^N_{\mathrm{PD}} \circ \cdots \circ \mathcal{E}^2_{\mathrm{PD}} \circ \mathcal{E}^1_{\mathrm{PD}}(\rho)$, where $\mathcal{E}^i_{\mathrm{PD}}(\rho) = (1-\xi_i)\,\rho + \xi_i \sigma_z^i \rho \sigma_z^i$, $\xi_i = \frac{1}{2}\left[1 - \exp\left(-\Delta t/T_2^i\right)\right]$ with T_2^i as the transversal relaxation time of the ith spin. The influence of the generalized amplitude damping can be approximately characterized as $\rho \to \mathcal{E}^N_{\mathrm{GAD}} \circ \cdots \circ \mathcal{E}^2_{\mathrm{GAD}} \circ \mathcal{E}^1_{\mathrm{GAD}}(\rho)$, where $\mathcal{E}^j_{\mathrm{GAD}}(\rho) = \sum_s E_s^j \rho E_s^{j\dagger}$,

$$\begin{aligned} E_1^j &= \sqrt{\frac{1}{2}}\begin{pmatrix} 1 & 0 \\ 0 & \sqrt{1-\eta_j} \end{pmatrix}, \quad E_2^j = \sqrt{\frac{1}{2}}\begin{pmatrix} 0 & 0 \\ \sqrt{\eta_j} & 0 \end{pmatrix}, \\ E_3^j &= \sqrt{\frac{1}{2}}\begin{pmatrix} \sqrt{1-\eta_j} & 0 \\ 0 & 1 \end{pmatrix}, \quad E_4^j = \sqrt{\frac{1}{2}}\begin{pmatrix} 0 & \sqrt{\eta_j} \\ 0 & 0 \end{pmatrix}, \end{aligned} \tag{S22}$$

$\eta_j = 1 - \exp\left(-\Delta t/T_1^j\right)$ with T_1^j as the longitudinal relaxation time of the jth qubit. The relaxation time for ^{31}P nuclear spin and ^{1}H nuclear spins are $T_2^{\mathrm{P}} = 1.30$ sec,$T_1^{\mathrm{P}} = 5$ sec and $T_2^{\mathrm{H}} = 1.26$ sec,$T_1^{\mathrm{H}} = 4.2$ sec, respectively. Moreover, a main source of phase damping in NMR experiment is the inhomogeneity of static magnetic field, and we suppress this effect by employing the refocusing sequences during the free evolution. The evolution time in a single experiment is 187 msec, thus the effect of the relaxation is not negligible. The numerical results show that the relative error of $\mathcal{L}_\delta^\Delta$ caused by the relaxation is 8.2%. To compensate for signal decay caused by relaxation during the evolution, we additionally measure a reference signal $S_0(\boldsymbol{\theta}) = Tr[V_0(\boldsymbol{\theta})\rho V_0^\dagger(\boldsymbol{\theta})\sigma_z]$ with $V_0(\boldsymbol{\theta}) = U_E^\dagger(\boldsymbol{\theta})U_E(\boldsymbol{\theta})$. Since the decay levels of $S^{\exp}(\boldsymbol{\theta})$ (experimental measurement of $S(\boldsymbol{\theta})$) and $S_0^{\exp}(\boldsymbol{\theta})$ (experimental measurement of $S_0(\boldsymbol{\theta})$) are approximately the same due to the similarity in evolutions $V_\delta(\boldsymbol{\theta})$ and $V_0(\boldsymbol{\theta})$, we use the following relation for calibration:

$$\frac{S^{\exp}(\boldsymbol{\theta})}{S(\boldsymbol{\theta})} \approx \frac{S_0^{\exp}(\boldsymbol{\theta})}{S_0(\boldsymbol{\theta})}.$$

Note that $V_0(\boldsymbol{\theta}) = \mathbb{1}$, so $S_0(\boldsymbol{\theta})$ does not depend on $\boldsymbol{\theta}$ and can be written as a known constant S_0. Consequently, we have the calibrated value for the target measurement $S(\boldsymbol{\theta})$ as

$$S(\boldsymbol{\theta}) = \frac{S_0(\boldsymbol{\theta})}{S_0^{\exp}(\boldsymbol{\theta})} \times S^{\exp}(\boldsymbol{\theta}).$$

TABLE S1: Analysis of the experimental errors and the experimental techniques employed to suppress the errors.

Totoal experimental error: 1.35%			
Sources of experimental error	Pulse error	Relaxation	Measurement error
Contributions (regular pulse sequence)	8.1%	8.2%	2.5×10^{-2}%
Contributions (specific experimental technique)	4.5×10^{-2}% (BB1 sequence)	0.98% (calibration with $\mathcal{L}_0^{\Delta}$)	1.8×10^{-2}% (composite pulse decoupling)

Following the calibration procedure outlined above, we conducted numerical simulations based on the relaxation dynamics described in Eq. (S22), demonstrating that the relative error can be reduced to 0.98%.

3. **Measurement error**: The systematic errors of pulse and relaxation lead to consistent deviation from the theoretical expectation in the experiment. While the measurement error comes from the stochastic fluctuations on NMR signal and thus corresponds to the error bound of repetitive measurements. Its effect can be calibrated from the signal-to-noise ratio (SNR) of the NMR spectra. To improve the SNR of the ^{31}P channel, protons are decoupled with composite pulses when measuring the signal of ^{31}P nuclear. According to the numerical simulation, the relative error of $\mathcal{L}_\delta^{\Delta}$ caused by the measurement error decreases from 2.5×10^{-2}% to 1.8×10^{-2}% after applying the decoupling operation. The absolute number of experimental error bounds can be analyzed via linear error propagation. For any linear function $f(x_1,x_2,...x_j,...)=\sum_j c_jx_j$, the variance is obtained from

$$\sigma_f^2=\sum_j\left|\frac{\partial f}{\partial x_j}\right|^2\sigma_{x_j}^2, \tag{S23}$$

where σ_f is the standard deviation of the function f, σ_{x_j} is the standard deviation of x_j. The experimental results of $\mathcal{L}_\delta^{\Delta}$ are obtained from the linear function $\mathcal{L}_\delta^{\Delta}\equiv\sum_{j=1}^{10}\gamma_j\mathrm{Tr}\left(V_\delta(\boldsymbol{\theta})\rho_{\mathrm{eq}}^{\Delta}V_\delta^{\dagger}(\boldsymbol{\theta})\sigma_z^j\right)$. In this case,$f=\mathcal{L}_\delta^{\Delta},|\partial f/\partial x_j|^2=\gamma_j^2$ and $x_j=\mathrm{Tr}\left(V_\delta(\boldsymbol{\theta})\rho_{\mathrm{eq}}^{\Delta}V_\delta^{\dagger}(\boldsymbol{\theta})\sigma_z^j\right)$ is the experimental measurement. The variance of NMR measurement can be calibrated from the SNR in the readout. Due to the polarization of nine identical ^{1}H spins can be obtained in a single measurement, we have $\sigma_{x_1}^2=0.32,\sum_{j=2}^{10}\sigma_{x_j}^2=0.02$. Hence the standard deviation of experimental $\mathcal{L}_\delta^{\Delta}$ is 1.06 and is much smaller than the amplitude of $\mathcal{L}_\delta^{\Delta}\sim10^4$.

The analysis above is summarized in Table. S1, which shows that the relaxation contributes to 0.98% relative error and is the main source of errors. This result is close to the experimental error of 1.35%.

VII. 'TIME-REVERSAL-BASED READOUT' PROTOCOL AND ITS PRECISION BOUND

The QFI decides the ultimate potential of quantum state, while this bound can only be saturated under optimal measurements. For an arbitrary nonclassical quantum probe, finding optimal measurements is challenging. Inspired by the fact that the 'time-reversal-based readout' can saturate the QCRB for arbitrary pure state [3], we generalize this protocol for mixed probe state. Though this protocol doesn't generate optimal measurements, it virtually saturates the QFI under specific parameters.

The 'time-reversal-based readout' protocol is realized by time reversing the engineering operation U_{E} and then project onto the initial state. Consequently, it's similar with the protocol for measuring LE except for replacing the quench $e^{-i\delta G}$ with the realistic encoding process $e^{-i\alpha G}$, where α is the unknown parameter. The precision for estimating α is given by standard error propagation

$$(\Delta\alpha)^2=\frac{(\Delta\mathcal{O})^2}{(d\langle\mathcal{O}\rangle/d\alpha)^2}, \tag{S24}$$

where $(\Delta\mathcal{O})^2:=\langle\mathcal{O}^2\rangle-\langle\mathcal{O}\rangle^2$. For the pure encoded state $e^{i\alpha G}|\Psi_f\rangle$, the QCRB is saturated when $\alpha\to0$ [3, 4],

$$(\Delta\alpha)^2=\left.\frac{(\Delta\mathcal{O}_{\mathrm{rev}})^2}{(d\langle\mathcal{O}_{\mathrm{rev}}\rangle/d\alpha)^2}\right|_{\alpha=0}=\frac{1}{\sqrt{\mathcal{F}(|\Psi_f\rangle)}}. \tag{S25}$$

Here $\mathcal{O}_{\mathrm{rev}}=|\Psi_f\rangle\langle\Psi_f|$ and we use the results

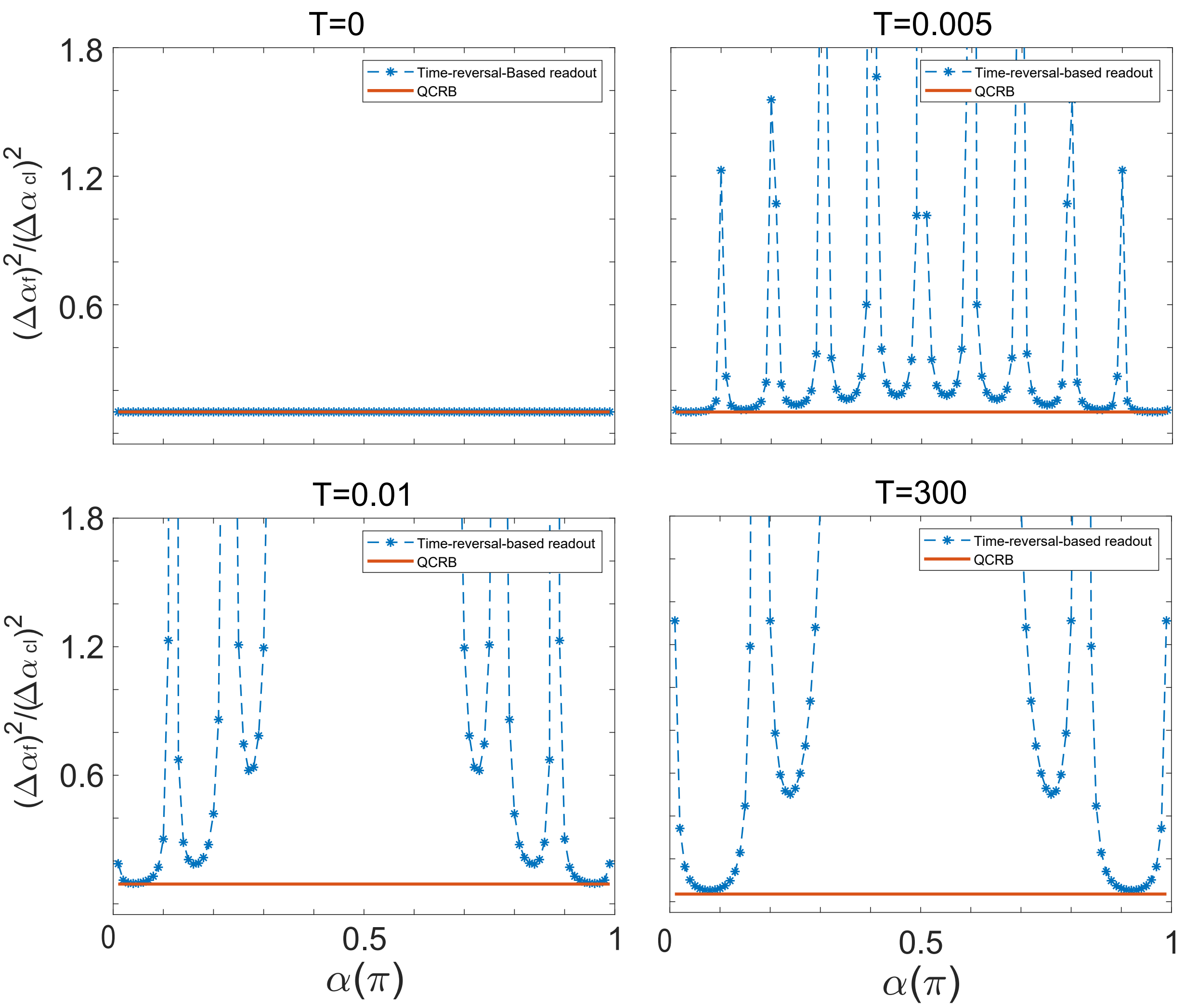

FIG. S6: The ratio of the precision obtained from our 'time-reversal-based readout' protocol to that of standard quantum limit under different purities. Here the purity depends on the Boltzmann distribution at specific temperature. When the temperature is 0K, the equilibrium state is pure and the precision of 'time-reversal-based readout' protocol can saturate the QCRB. The performance of this protocol becomes worse as the increase of temperature, while a precision close to QCRB is still attainable on some specific region of α even at room temperature.

$$
\begin{aligned}
\Delta\mathcal{O}_{\rm rev} =& \langle(|\Psi_f\rangle\langle\Psi_f|)^2\rangle - \langle|\Psi_f\rangle\langle\Psi_f|\rangle^2 \\
=& \frac{\alpha^2}{4}\mathcal{F}(|\Psi_f\rangle) + O(\alpha^4) \\
\langle\mathcal{O}_{\rm rev}\rangle =& 1 - \frac{\alpha^2}{4}\mathcal{F}(|\Psi_f\rangle) + O(\alpha^4),
\end{aligned}
\tag{S26}
$$

where the expectation is taken over the encoded probe, i.e., $\langle\cdot\rangle := \langle\Psi_f|e^{i\alpha G}\cdot e^{-i\alpha G}|\Psi_f\rangle$.

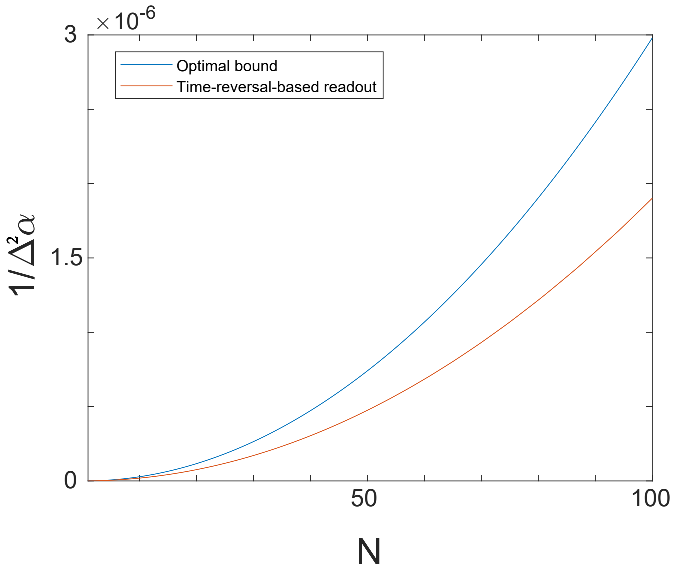

FIG. S7: The scaling of sensitivity under the 'time-reversal-based readout' protocol (red solid line) in terms of the number of particles when $\epsilon = 10^{-5}$. It can still achieve the Heisenberg scaling with a ratio to the optimal bound given by Ref. [2] (blue solid line) of 0.638.

For the case of mixed probe, the precision $\Delta\alpha$ can also be obtained from Eq. (S24) by calculating $\langle \mathcal{O}_{\rm rev}\rangle$ and $\langle \mathcal{O}^2_{\rm rev}\rangle$,

$$\begin{aligned}\langle \mathcal{O}_{\rm rev}\rangle =&\mathrm{Tr}\left(e^{-i\alpha G}\rho_{\rm f}e^{i\alpha G}\rho_{\rm f}\right)\\ =&\mathrm{Tr}\left(\sum_{m_1}\rho_{m_1}\sum_{m_2}\rho_{m_2}e^{-im_2\alpha}\right)\\ =&\sum_m \mathrm{Tr}\left(\rho_{-m}\rho_m\right)e^{-im\alpha}\\ \langle \mathcal{O}^2_{\rm rev}\rangle =&\mathrm{Tr}\left(e^{-i\alpha G}\rho_{\rm f}e^{i\alpha G}\rho_{\rm f}^2\right)\\ =&\sum_{m_1,m_2}\mathrm{Tr}\left(\rho_{m_1}\rho_{-m_1-m_2}\rho_{m_2}e^{-im_2\alpha}\right),\end{aligned} \tag{S27}$$

where we divide the density matrix of probe into blocks as $\rho_{\rm f}=\sum_m\sum_{\lambda_i-\lambda_j=m}\rho_{ij}|i\rangle\langle j|=\sum_m\rho_m$ with $G|i\rangle=\lambda_i|i\rangle$ [5]. In the following, we specifically consider the initial probe ρ_0 in thermal equilibrium, which is close to the case in our experiment. The equilibrium state can be expressed as

$$\rho_0=(\lambda_0|0\rangle\langle 0|+\lambda_1|1\rangle\langle 1|)^{\otimes N}, \tag{S28}$$

where $\lambda_0=\frac{e^{\hbar\omega/k_{\rm B}T}}{e^{\hbar\omega/k_{\rm B}T}+e^{-\hbar\omega/k_{\rm B}T}}, \lambda_1=\frac{e^{-\hbar\omega/k_{\rm B}T}}{e^{\hbar\omega/k_{\rm B}T}+e^{-\hbar\omega/k_{\rm B}T}}$, $k_{\rm B}=1.38\times10^{-23}\mathrm{JK}^{-1}$ is the Boltzmann constant, and $|\hbar\omega|=2.6\times10^{-25}$J is the energy difference between the Zeeman states for the case of protons in a field of 9.4T [6]. For the optimal probe state engineered by unitary operation from ρ_0 (given by Eq. (S8)), the expression of $\langle \mathcal{O}_{\rm rev}\rangle$ and $\langle \mathcal{O}^2_{\rm rev}\rangle$ become

$$\begin{aligned}\langle \mathcal{O}_{\rm rev}\rangle =&\sum_{i=0}^{\lfloor\frac{N}{2}\rfloor}C_N^i\left\{\frac{1}{2}\left(\lambda_0^{N-i}\lambda_1^i+\lambda_0^i\lambda_1^{N-i}\right)+\frac{1}{2}\cos[(N-2i)\alpha]\left(\lambda_0^{N-i}\lambda_1^i-\lambda_0^i\lambda_1^{N-i}\right)\right\},\\ \langle \mathcal{O}^2_{\rm rev}\rangle =&\sum_{i=0}^{\lfloor\frac{N}{2}\rfloor}C_N^i\frac{1}{2}\left[\left(\lambda_0^{N-i}\lambda_1^i\right)^2+\left(\lambda_0^i\lambda_1^{N-i}\right)^2\right]\left(\lambda_0^{N-i}\lambda_1^i+\lambda_0^i\lambda_1^{N-i}\right)\\ &C_N^i\frac{1}{2}\cos[(N-2i)\alpha]\left[\left(\lambda_0^{N-i}\lambda_1^i\right)^2-\left(\lambda_0^i\lambda_1^{N-i}\right)^2\right]\left(\lambda_0^{N-i}\lambda_1^i-\lambda_0^i\lambda_1^{N-i}\right),\end{aligned} \tag{S29}$$

respectively, according to Eq. (S27). Here C_N^i represents the binomial coefficient. In Fig. S6, we show the precision ratio of optimal engineered state under the 'time-reversal-based readout' protocol to that of classical uncorrelated state

in Sec. III under different temperatures. When the temperature is 0K, the equilibrium state is pure and the precision under the 'time-reversal-based readout' protocol can saturate the QCRB. The performance of this protocol becomes worse as the increase of temperature, while a precision close to QCRB is still attainable even at room temperature, i.e., the case of our experiments. In this way, we can set the working point as $\widetilde{\alpha} = \mathrm{argmin}_{\alpha}\Delta\alpha$ for the best metrological performance. The encoded α is priori unknown, while it can be shifted to $\widetilde{\alpha}$ with the adaptive method to saturate the local precision limit [7, 8].

We further investigate the scaling of the sensitivity of the 'time-reversal-based readout' protocol. Here, the precision bound is obtained at the optimal point $\widetilde{\alpha}$. As shown in Fig. S7, the Heisenberg scaling in terms of the number of particles can still be obtained, whose ratio to the optimal bound in Ref. [2] is 0.638.

[1] W. Spendley, G. R. Hext, and F. R. Himsworth, Technometrics **4**, 441 (1962).
[2] K. Modi, H. Cable, M. Williamson, and V. Vedral, Physical Review X **1**, 021022 (2011).
[3] S. P. Nolan, S. S. Szigeti, and S. A. Haine, Phys Rev Lett **119**, 193601 (2017).
[4] T. Macrì, A. Smerzi, and L. Pezzè, Physical Review A **94**, 010102 (2016).
[5] M. Garttner, P. Hauke, and A. M. Rey, Phys Rev Lett **120**, 040402 (2018).
[6] M. H. Levitt, *Spin dynamics: basics of nuclear magnetic resonance* (John Wiley and Sons, 2013).
[7] R. D. GILL, Conciliation of bayes and pointwise quantum state estimation, in *Quantum Stochastics and Information*, pp. 239–261.
[8] A. Fujiwara, Journal of Physics A: Mathematical and General **39**, 12489 (2006).
